# Integrated multi-omics elucidates the dual anti-inflammatory and neuroendocrine mechanisms of novel TCM plasters against primary dysmenorrhea

**DOI:** 10.3389/fpain.2026.1727963

**Published:** 2026-03-11

**Authors:** Weihui Liang, Wenxuan Cao, Yuan Zheng, Tie Li, Aoxue Yu, Fuchun Wang, Jia Liu

**Affiliations:** 1 Department of Pharmaceutical Science, Changchun University of Chinese Medicine, Changchun, China; 2 Department of Clinical Pharmacy, The First Hospital of Jilin University, Changchun, China; 3 Department of Acupuncture and Massage, Changchun University of Chinese Medicine, Changchun, China

**Keywords:** estrogen signaling pathway, hypothalamic-pituitary-ovarian axis, inflammation, multi-omics analysis, primary dysmenorrhea

## Abstract

**Background:**

Primary dysmenorrhea (PD) is a prevalent gynecological condition primarily driven by uterine inflammation and hormonal imbalances. While non-steroidal anti-inflammatory drugs (NSAIDs) are the first-line treatment, their side effects and failure rate necessitate alternative therapies. The Nuangong Zhitong Plaster (NGZT) and its graphene-modified variant (SMX) are clinically used traditional Chinese medicine (TCM) formulations for PD, but their comprehensive mechanisms of action remain unclear.

**Methods:**

A rat PD model was established via estradiol benzoate and oxytocin injection. Rats were treated with NGZT, SMX, ibuprofen, or a loxoprofen patch. Therapeutic effects were assessed through pain behavior scoring, uterine coefficient measurement, and enzyme-linked immunosorbent assay (ELISA) for prostaglandins and β-endorphin. An integrated approach combining network pharmacology, transcriptomics, proteomics, and metabolomics was employed to uncover the mechanisms, followed by experimental validation using Western blot, reverse transcription quantitative polymerase chain reaction (RT-qPCR), and coagulation function tests.

**Results:**

Both NGZT and SMX significantly alleviated pain behaviors, reduced uterine swelling, and normalized levels of pain mediators. Network pharmacology and molecular docking predicted multi-target binding against core proteins such as prostaglandin-endoperoxide synthase 2 (PTGS2/COX-2), estrogen receptor 1 (ESR1), and cytochrome P450 family 19 subfamily A member 1 (CYP19A1). Multi-omics analyses revealed that the plasters reversed PD-associated alterations by co-regulating arachidonic acid metabolism and estrogen signaling pathways. Experimental validation confirmed that the plasters suppressed uterine expression of COX-2, interleukin-6 (IL-6), and interleukin-1β (IL-1β), corrected systemic hypercoagulability, and restored sex hormone balance. This was achieved through modulation of key components within the hypothalamic-pituitary-ovarian (HPO) axis—specifically gonadotropin-releasing hormone (GnRH) and its receptor (GnRH-R)—as well as uterine hormone receptors including the estrogen receptor (ER), progesterone receptor (PR), and oxytocin receptor (OTR). SMX demonstrated superior efficacy in modulating certain inflammatory and hormonal parameters, likely attributable to enhanced transdermal delivery facilitated by graphene.

**Conclusion:**

This study provides the comprehensive evidence that NGZT and SMX exert their anti-dysmenorrhea effects through a synergistic, multi-target mechanism that concurrently dampens uterine inflammation and rebalances the neuroendocrine system. The integration of multi-omics offers a robust systems-level framework for deciphering the mechanism of complex TCM formulations, solidifying the scientific basis for their clinical application in pain management.

## 1 Introduction

Primary dysmenorrhea (PD), characterized by cramping pain in the lower abdomen occurring shortly before or during menstruation in the absence of pelvic pathology, represents one of the most prevalent gynecological disorders among women of reproductive age (1, 2). Its significant negative impact on quality of life, productivity, and daily activities is often underestimated due to the common misconception that menstrual pain is a normal physiological phenomenon, leading to substantial under-diagnosis (3, 4). Epidemiological studies reveal a strikingly high prevalence, affecting 45%–95% of women, with some reports indicating that up to 93% of female high school students in Australia experience PD (5, 6). These figures underscore the urgent need for developing effective and well-tolerated therapeutic strategies.

The pathogenesis of PD is primarily attributed to the elevated secretion of prostaglandins (PGs), particularly prostaglandin F2 alpha (PGF2α), in the endometrium during menstruation, which induces intense uterine contractions and ischemia (7, 8). Consequently, nonsteroidal anti-inflammatory drugs (NSAIDs), which inhibit the cyclooxygenase (COX) enzymes responsible for PG synthesis, constitute the first-line conventional therapy (9). However, their utility is constrained by a 20%–25% treatment failure rate and a spectrum of adverse effects, including gastrointestinal discomfort, bleeding, and dizziness (10, 11). This clinical dilemma has fueled the search for alternative treatments, among which Traditional Chinese Medicine (TCM) has garnered attention for its holistic approach and perceived safety profile (12).

Within the TCM arsenal, acupoint sticking therapy offers a unique non-invasive option. It facilitates drug delivery through the skin, potentially bypassing hepatic first-pass metabolism and providing sustained release (13). The Nuangong Zhitong Acupoint Plaster (NGZT) is a composite formulation of 13 medicinal herbs based on the TCM principle of “warming the palace and relieving pain”. To further enhance delivery, a graphene-modified variant (SMX) was engineered. Graphene's excellent thermal conductivity and large surface area are hypothesized to improve the release and transdermal penetration of active constituents (14, 15). Preliminary clinical observations suggest the efficacy of both NGZT and SMX, especially when combined with other TCM modalities (16, 17). Nevertheless, a comprehensive and systematic investigation into their underlying pharmacological mechanisms remains lacking, hindering their broader scientific acceptance.

The complex pathophysiology of PD, involving hormonal fluctuations, inflammatory cascades, and oxidative stress, necessitates a research approach that can capture this multi-faceted nature (1). The advent of omics technologies—including transcriptomics, proteomics, and metabolomics—provides an unparalleled platform for unbiased, system-level exploration of disease mechanisms and drug actions (18). While these techniques have been individually applied in PD research (19–22), their integrated application to decipher the mechanisms of complex TCM formulations like NGZT and SMX is still in its infancy. A multi-omics integration strategy can bridge the gap between molecular perturbations and phenotypic outcomes, offering a holistic understanding of therapeutic effects.

**Figure 1:**
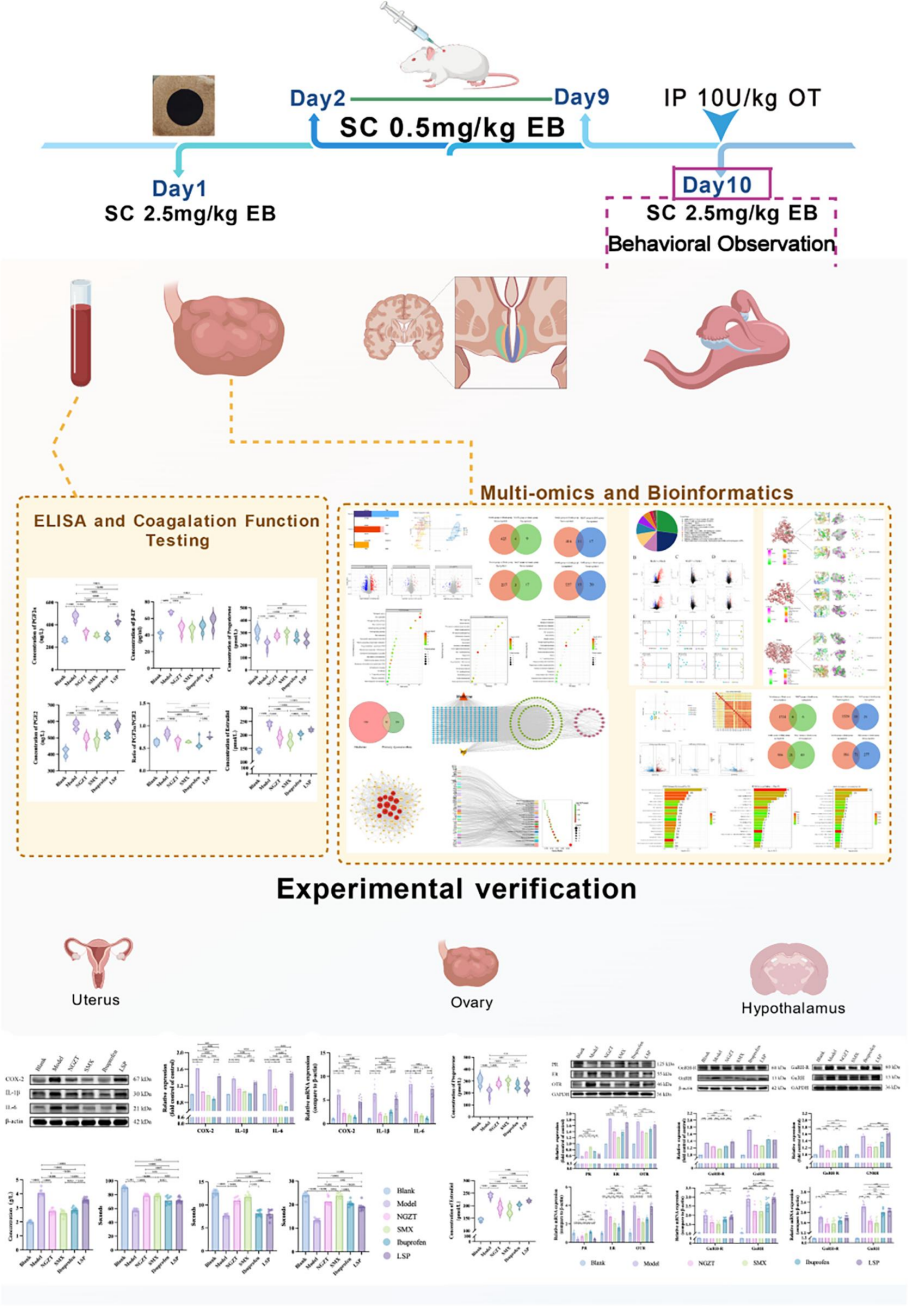
Study design flowchart. The Primary dysmenorrhea (PD) model was induced by subcutaneous (s.c.) injection of estradiol benzoate (EB) at a dosage of 2.5 mg/kg on days 1 and 10, and 0.5 mg/kg on days 2–9. This was followed by an intraperitoneal (i.p.) injection of oxytocin (OT) at a dosage of 10 U/kg on day 10. Pain-related behaviors were assessed on day 10. Following sample collection, integrated multi-omics analyses, including transcriptomics, proteomics, and metabolomics, along with bioinformatics analyses, were conducted, followed by experimental validation using molecular and biochemical assays. The key tissues analyzed included the uterus, ovary, and hypothalamus. Timeline, rat, syringe, blood collection tubes, ovary, brain, and uterus created with https://www.BioGDP.com (23).

Therefore, this study was designed to elucidate the efficacy and multi-target mechanisms of NGZT and SMX in a rat model of PD. We employed an integrated approach combining pharmacodynamic assessments with transcriptomic, proteomic, and metabolomic analyses, followed by bioinformatic integration and experimental validation. We hypothesized that the therapeutic effects of these plasters are mediated through the coordinated modulation of key pathways involved in inflammation, sex hormone balance, and the hypothalamic-pituitary-ovarian (HPO) axis. The overall design of this study is summarized in Figure 1 [Created with https://www.BioGDP.com (23)].

## 2 Materials and methods

### 2.1 Animals and ethical statement

A total of sixty specific-pathogen-free (SPF) female Sprague Dawley rats (weighing 200 ± 20 g) were obtained from Changchun Yisi Laboratory Animal Technology Co. Ltd. [Certificate SCXK(Ji)-2020-0002]. The rats were housed under standard laboratory conditions with a 12 h light/dark cycle, ambient temperature of 23 °C ± 2 °C, and relative humidity of 55% ± 10%. They were provided with standard rodent chow and water *ad libitum*. All experimental procedures were approved by the Animal Care and Use Committee of Changchun University of Chinese Medicine (Approval No: 2022005) and were conducted in accordance with the National Institutes of Health guide for the care and use of Laboratory animals.

### 2.2 Experimental design and drug administration

After one week of acclimatization, the rats were randomly assigned to six groups (*n* = 10 per group): Blank group: Received no modeling induction and only abdominal wrapping; Model group: Subjected to the PD model induction and abdominal wrapping; NGZT group: Modeled and treated with the Nuangong Zhitong Plaster; SMX group: Modeled and treated with the graphene-modified Nuangong Zhitong Plaster; Ibuprofen group: Modeled and treated with oral ibuprofen; Loxoprofen Sodium Patch (LSP) group: Modeled and treated with the loxoprofen transdermal patch.

Drugs and Dosing: NGZT and SMX: The plasters were provided by Jilin Guoda Biological Engineering Co. Ltd. (Cat. No.: 20210524 and 20220801, respectively). The plaster was applied to the shaved skin of the Shenque acupoint (RN8, umbilicus) and secured with surgical tape. The plaster was replaced every 24 h for 10 consecutive days; Ibuprofen: (Harbin Pharmaceutical Group, Cat. No.: 22024074) was suspended in carboxymethyl cellulose sodium (CMC-Na) solution and administered by oral gavage at a dose of 0.02 g/kg once daily for 10 days; LSP: (Daiichi Sankyo, Cat. No.: L041R) was applied to the shaved abdominal skin (away from the Shenque acupoint) following the manufacturer's instructions and replaced every 24 h.

### 2.3 Primary dysmenorrhea model induction

The PD model was established according to a previously published method (22) with minor modifications. Briefly, rats in all groups except the Blank group received subcutaneous (s.c.) injections of estradiol benzoate (EB; Harbin Sanma Veterinary Pharmaceutical Co. Ltd., Cat. No. 20210801) once daily for 10 days. The dosage regimen was as follows: 2.5 mg/kg on days 1 and 10, and 0.5 mg/kg on days 2–9. Concurrently with the EB injections, the medicated plasters or abdominal wrappings were applied as described above.

On the final day, 1 h after the last EB injection and treatment application, all rats except those in the Blank group received an intraperitoneal (i.p.) injection of oxytocin (OT; Jilin Huamu Animal Health Products Co. Ltd., Cat. No. 20210524) at 10 U/kg to induce uterine contraction and writhing response.

### 2.4 Pain behavior assessment

Immediately following the OT injection, each rat was placed individually in a transparent observation cage. Their behavior was scored over a 30 min period by two independent observers who were blinded to the group allocation. The writhing response was quantified using a validated scoring system (24), with minor adaptations. The scoring criteria were defined as follows: 0 points for normal behavior without writhing; 1 point for body leaning to either side or arching of the back sustained for approximately 1 min; 2 points for full extension of the hind limbs with the body lying flat on the cage bottom, combined with a sideways rotation of the pelvis; and 3 points for intense abdominal muscle contractions accompanied by body extension and rotation (as detailed in Table 1). The total writhing score for each animal was calculated as the sum of points recorded throughout the entire observation period.

**Table 1:** Criteria for scoring writhing behavior in rats.

Score	Behavior
0	Normal behavior, no writhing
1	Leaning body to the left or right
	Arching back for 1 min
2	Hind limbs and body extended and flat on the bottom, pelvis rotated sideways
3	Abdominal muscle contraction, body extension and rotation

### 2.5 Sample collection

Following the behavioral observation, all rats were anesthetized by i.p. injection of 2% pentobarbital sodium (30 mg/kg). Blood was collected from the abdominal aorta. For plasma preparation, blood was mixed with sodium citrate (1:9, v/v) and centrifuged at 3,000 rpm for 15 min. For serum, blood was allowed to clot at room temperature for 30 min prior to centrifugation. The uteri, ovaries, and hypothalami were rapidly dissected. The wet weight of each uterus was recorded to calculate the uterine coefficient [uterine weight (g)/body weight (g)]. Each uterus was subsequently divided into two parts: one part was snap-frozen in liquid nitrogen for multi-omics analyses, while the other was stored at −80 °C for molecular biology assays. The ovaries and hypothalami were also stored at −80 °C for further analysis.

### 2.6 Coagulation function tests

Citrated plasma was used for coagulation tests. The concentrations of fibrinogen (Fib), thrombin time (TT), prothrombin time (PT), and activated partial thromboplastin time (APTT) were determined using an automatic multifunctional biochemical analyzer (SMT-120VP, Seamaty, China) according to the manufacturer's protocols.

### 2.7 Enzyme-Linked immunosorbent assay (ELISA)

The concentrations of prostaglandin F2α (PGF2α), prostaglandin E2 (PGE2), β-endorphin (β-EP), progesterone, and estradiol in serum or plasma were quantified using commercial ELISA kits (Jiangsu Meimian Industrial Co. Ltd., China) strictly following the manufacturer's instructions. The absorbance was measured at 450 nm using a multi-function microplate reader (BioTek, USA).

### 2.8 Western blot analysis

Total protein was extracted from uterine, ovarian, and hypothalamic tissues using RIPA lysis buffer supplemented with protease and phosphatase inhibitors. Protein concentration was determined using a bicinchoninic acid (BCA) assay kit (Beyotime, China). Equal amounts of protein (80 μg per lane) were separated by sodium dodecyl sulfate-polyacrylamide gel electrophoresis (SDS-PAGE) and transferred onto polyvinylidene difluoride (PVDF) membranes (Millipore, USA). After blocking with 5% non-fat milk, the membranes were incubated overnight at 4 °C with primary antibodies against interleukin-6 (IL-6, 1:1000, Wanlei, WL02841), interleukin-1β (IL-1β, 1:1000, Affinity, AF5103), progesterone receptor (PR, 1:1000, Bioss, bs-23376R), cyclooxygenase-2 (COX-2, 1:2000, Proteintech, 66351-1-Ig), estrogen receptor (ER, 1:2000, Proteintech, 21244-1-AP), oxytocin receptor (OTR, 1:1500, Proteintech, 23045-1-AP), gonadotropin-releasing hormone receptor (GnRH-R, 1:1500, Proteintech, 22462-1-AP), and gonadotropin-releasing hormone (GnRH, 1:1000, Abclonal, A5625). β-actin (1:5000, TransGen, Q10410) or GAPDH (1:5000, TransGen, Q10614) served as the loading control. After incubation with horseradish peroxidase (HRP)-conjugated secondary antibodies, protein bands were visualized using an enhanced chemiluminescence (ECL) reagent (Mona, China) and quantified using ImageJ software (v2.3.0).

### 2.9 Quantitative real-time PCR (RT-qPCR)

Total RNA was extracted using the RNA simple Total RNA Kit (Tiangen, China). Complementary DNA (cDNA) was synthesized using the FastKing gDNA Dispelling RT SuperMix (Tiangen). RT-qPCR was performed on a CFX96 system (Bio-Rad, USA) using SuperReal PreMix Plus (SYBR Green) (Tiangen). The primer sequences are listed in Table 2. The relative mRNA expression levels were calculated using the 2^(^−ΔΔCt^) method, with β-actin as the internal control.

**Table 2:** Primer sequences used for quantitative real-time PCR (RT-qPCR).

Gene name	Forward sequence (5′→3′)	Reverse sequence (5′→3′)
IL-6	CACTGGTCTTTTGGAGTTTGAG	GGACTTTTGTACTCATCTGCAC
IL-1β	AATCTCACAGCAGCATCTCGACAAG	TCCACGGGCAAGACATAGGTAGC
COX-2	ACACTCTATCACTGGCATCC	GAAGGGACACCCTTTCACAT
PR	GTCCCCAGTTCACAACGCTTC	GGGCAGCAATAACTTCAGACATC
ER	CAGGCTTTGGGGACTTGAATCT	TGATTCCTGTCCAAGAGCAAGTTAG
OTR	CTTCTGCTGCTCTGCTCGTTACC	TGGATGAGTTGCTCTTCTTGCTGAC
GnRH	GCCGCTGTTGTTCTGTTGACTGTG	GAAGTTCTGGGGTTCTGCCATTTG
GnRH-R	TTGTCTTTGCGGGACCACAGTTAT	GTGGGTCACACATTGCGAGAAAAC
β-actin	CTGGCACCACACCTTCTACAATGAG	GATAGCACAGCCTGGATAGCAACG

### 2.10 Bioinformatics analysis

#### 2.10.1 Target prediction and network construction

The active components of NGZT, a formulation comprising *Cyperi Rhizoma* (Xiangfu), *Caryophylli Flos* (Dingxiang), *Corydalis Rhizoma* (Yanhusuo), *Olibanum* (Ruxiang), *Myrrha* (Moyao), *Angelicae Sinensis Radix* (Danggui), *Angelicae Dahuricae Radix* (Baizhi), *Leonuri Herba* (Yimucao), *Carthami Flos* (Honghua), *Foeniculi Fructus* (Huixiang), *Cinnamomi Ramulus* (Guizhi), *Zingiberis Rhizoma* (Ganjiang), and *Paeoniae Radix Alba* (Baishao), were retrieved from the Traditional Chinese Medicine Systems Pharmacology Database (25) (TCMSP, https://tcmspw.com/). Components with a drug-likeness (DL) ≥0.18 were selected. Their potential targets were predicted using the SwissTargetPrediction database (26) (http://www.swisstargetprediction.ch/).

PD-related targets were collected from DisGeNET (27) (https://www.disgenet.org/), OMIM (28) (https://omim.org/), and GeneCard (29) (https://www.genecards.org/) databases using “primary dysmenorrhea” as the keyword. The common targets between NGZT and PD were identified and a compound-target-pathway network was visualized using Cytoscape software (v3.7.2).

#### 2.10.2 Protein-Protein interaction (PPI) and enrichment analysis

The common targets were imported into the STRING database (30) (https://string-db.org/) to construct a PPI network. This network was generated using the following parameters: the organism was set to *Homo sapiens*, a full STRING network type was selected, the minimum required interaction score was established at high confidence (0.700), and the false discovery rate (FDR) stringency was set to medium (5%). The resulting network was subsequently analyzed using the STRING platform and Cytoscape software to compute node connectivity. The top hub genes were identified based on their degree of connectivity within the network. Concurrently, Kyoto Encyclopedia of Genes and Genomes (KEGG) pathway enrichment analysis was performed for the common targets using the DAVID database (31) (https://david.ncifcrf.gov/). The enriched pathways were filtered based on a significance threshold of *p* < 0.05, ranked by the number of involved genes, and the top 20 most significant pathways were selected for subsequent visualization.

#### 2.10.3 Molecular docking

To validate the predicted interactions between key bioactive compounds and core targets, molecular docking simulations were conducted. Based on the core targets (CYP19A1, ESR1, and PTGS2) prioritized by the preceding network pharmacology analysis, their respective three-dimensional crystal structures were retrieved from the RCSB Protein Data Bank (PDB) database (32–34) (http://www.rcsb.org/). Specifically, the structure of human aromatase (CYP19A1) was obtained under the PDB ID 4GL7 (resolution: 3.90 Å), the ligand-binding domain of human estrogen receptor α (ESR1) under PDB ID 1QKU (resolution: 3.20 Å), and human prostaglandin-endoperoxide synthase 2 (PTGS2/COX-2) under PDB ID 5KIR (resolution: 2.70 Å). These structures were selected based on criteria of high resolution, determination by x-ray diffraction, and relevance to human homology. The downloaded PDB files were then preprocessed using PyMOL 2.4.0 and AutoDock 4.2.6. This involved removing solvent molecules and co-crystallized ligands, adding polar hydrogen atoms and computing Gasteiger charges, culminating in the export of the prepared protein structures in PDBQT format for subsequent docking. The SDF structures of the five selected ligand molecules (11-Keto-beta-boswellic acid, Eugenol, Imperatorin, Paeoniflorin, and Tetrahydropalmatine) were obtained from the PubChem database (35) (https://pubchem.ncbi.nlm.nih.gov/). OpenBabel 2.4.1 was utilized as a conversion tool to transform the small molecule ligands from SDF to PDB format. Subsequently, semi-flexible docking simulations between the active compounds and hub targets were performed using AutoDockTools 1.5.6 software. The results, representing the stable conformations of each ligand-receptor complex, were then imported into PyMOL 2.4.0 and Discovery Studio 4.5 for visual processing and analysis of the binding interactions.

### 2.11 Multi-omics data processing and analysis

#### 2.11.1 Transcriptomics analysis

##### 2.11.1.1 RNA extraction

Total RNA was extracted from uterine by using TRIzol reagent (Magen, China), and RNA samples were examined for concentration, purity, and integrity by Nanodrop (Thermo Fisher Scientific, USA) and Bioanalyz instruments (Agilent Technologies, No. 4150).

##### 2.11.1.2 Library construction

Paired-end (PE) libraries were prepared strictly according to the mRNA-seq Lib Prep Kit instructions (ABclonal, China). The mRNA was purified using oligo (dT) magnetic beads, and the mRNA fragments were used as templates to synthesize the second strand of cDNA. After the amplification by PCR, the quality of the libraries was next assessed by using Agilent Bioanalyzer. Finally, sequencing was performed using the NovaSeq sequencing platform (6000, Illumina, USA) to read length.

##### 2.11.1.3 Processing of data

HISAT2 (http://daehwankimlab.github.io/hisat2/) was used to sequence the clean reads against the specified genome to obtain information about its position on the reference genome. Subsequently, Deseq2 (http://bioconductor.org/packages/release/bioc/html/DESeq2.html) was used to analyze the differential expression of the genes, *p* < 0.05 and |log2[fold change (FC)]| > 1 were used as criteria for screening differentially expressed genes (DEGs).

#### 2.11.2 Proteomics analysis

##### 2.11.2.1 Protein extraction and peptide digestion

The samples were lysed by adding SDT lysis [4%(w/v) SDS, 100 mM Tris/HCl, pH 7.6]) to the uteri, and the supernatant was aspirated after centrifugation. The peptides were desalted and lyophilized after digesting, and then re-solubilized by adding 0.1% formic acid solution for subsequent separation and detection.

##### 2.11.2.2 Analysis by mass spectrometry and chromatography

Proteins were separated by high-performance liquid chromatography (HPLC) liquid phase system Easy nLC 1200 (Fisher Scientific, USA). Mobile phase A was 0.1% formic acid aqueous solution and mobile phase B was 0.1% formic acid acetonitrile aqueous solution (acetonitrile was 84%). After separating by chromatography, samples were analyzed by mass spectrometry by using a timsTOF Pro2 (Bruker, GER). The detection mode was positive ion and the ionization source voltage was set to 1.5 kV. The mass spectrometry scanning range was set to 100–1700 m/z and the analyzed precursor fragments were processed with a time-of-flight (TOF) detector and the data acquisition mode was parallel accumulation–serial fragmentation (PASEF) mode.

##### 2.11.2.3 Processing of data

The raw data were input into MaxQuant (v1.6.14) for library identification and quantitative analysis. *p* < 0.05 and |log2(FC)| > 1 were set as the criteria for screening differentially expressed proteins (DEPs).

#### 2.11.3 Metabolomics analysis

##### 2.11.3.1 Sample extraction

Appropriate amount of uterine was weighed and added to pre-cooled methanol/germanium/water solution (2:2:1, v/v/v). The mixture was then centrifuged and the supernatant was freeze-dried, finally stored them at −80 °C ambient. Aliquots of each sample are mixed to prepare quality control (QC) samples. For mass spectrometry analysis, 100 μl of acetonitrile water solution (1:1, v/v) was added to the freeze-dried supernatant, after vortexing and centrifuging, the supernatant is analyzed.

##### 2.11.3.2 Analysis by mass spectrometry and chromatography

The reconstituted samples were separated on a hydrophilic interaction liquid chromatography (HILIC) column of an ultrahigh-performance liquid chromatography system (1290 Infinity LC, Agilent, USA). The column temperature was 25℃, the flow rate was 0.5 ml/min, the injection volume was 2 μl, and the mobile phase A consisted of water, 25 mm ammonium beryllat and 25 mm ammonia. The mobile phase B consisted of acetonitrile. The gradient elution procedure is shown in Table 3. QC samples were inserted into the sample queue to monitor and evaluate system stability. After separating the sample, Triple TOF 6600 mass spectrometer (AB SCIEX, China) was used to analyze, and modes of ESI + and ESI- were used for detection respectively.

**Table 3:** Gradient elution procedure for hydrophilic interaction liquid chromatography (HILIC) in metabolomics analysis.

Time (min)	Mobile phase A (%)	Mobile phase B (%)
0–0.5	5	95
0.5–7	5–35	95–65
7–8	35–60	65–40
8–9	60	40
9–9.1	60–5	40–95
9.1–12	5	95

##### 2.11.3.3 Processing of data

The raw data were converted into mzXML format using the ProteoWizard platform, peak alignment, retention time correction and peak area extraction were performed by using XCMS software. In univariate analysis, metabolites with FC > 1.5, FC < 0.67 or *p* < 0.05 were regarded as the differential metabolites (DMs). In multidimensional statistical analysis, metabolites with a variable importance in projection (VIP) greater than 1 and a *p* less than 0.05 were utilized for subsequent enrichment analysis.

### 2.12 Statistical analysis

All data are presented as the mean ± standard deviation (SD). Statistical analysis was performed using GraphPad Prism software (v9.5.0). Differences between multiple groups were analyzed by one-way analysis of variance (ANOVA) followed by an appropriate *post hoc* test. A *p*-value of less than 0.05 was considered statistically significant.

## 3 Results

### 3.1 Both NGZT and SMX plasters ameliorate pain behaviors and key pain mediators in PD

To evaluate the therapeutic efficacy of the plasters, we first assessed pain-related behaviors and the levels of key pain mediators (prostaglandins and β-endorphin) in a rat model of PD.

**Figure 2:**
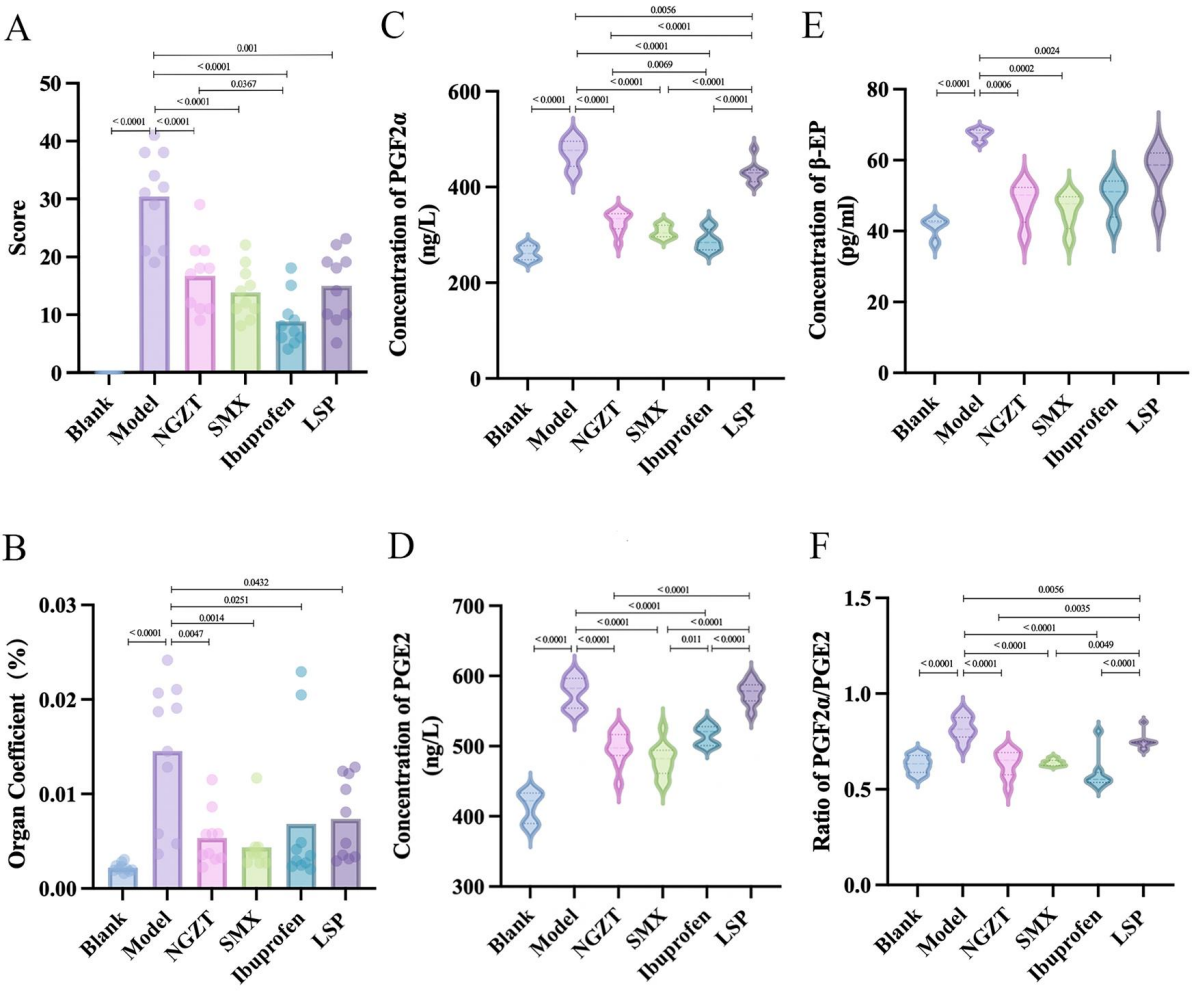
NGZT and SMX alleviate pain and normalize pain-related mediators in PD rats. **(A)** Writhing scores. **(B)** Uterine coefficient. **(C)** Serum prostaglandin F2α (PGF2α) levels. **(D)** Serum prostaglandin E2 (PGE2) levels. **(E)** Plasma β-endorphin (β-EP) levels. **(F)** PGF2α/PGE2 ratio. Statistical significance was determined using one-way analysis of variance (ANOVA) followed by Tukey's multiple comparisons test.

As illustrated in Figure 2A, rats in the model group exhibited significantly higher writhing scores compared to the blank group, confirming the successful establishment of the model. Treatment with NGZT, SMX, ibuprofen, and LSP all significantly reduced the writhing response, with ibuprofen demonstrating the most potent effect. Consistent with the behavioral data, uterine swelling, indicated by an increased uterine coefficient, was observed in the model group and was significantly attenuated by all treatments (Figure 2B).

At the biochemical level, the model group exhibited a pronounced dysregulation of pain and inflammatory mediators. Serum levels of PGF2α and PGE2, as well as the PGF2α/PGE2 ratio, were significantly elevated, while the analgesic factor β-EP in plasma was also increased, reflecting a compensatory response to pain (Figures 2C–F). Both TCM plasters and ibuprofen effectively reversed these changes, normalizing the levels of prostaglandins and β-EP. These results demonstrate that NGZT and SMX produce significant analgesic effects in PD, comparable to the positive control ibuprofen.

### 3.2 Network pharmacology predicts a multi-target mechanism and identifies core targets and compounds

**Figure 3:**
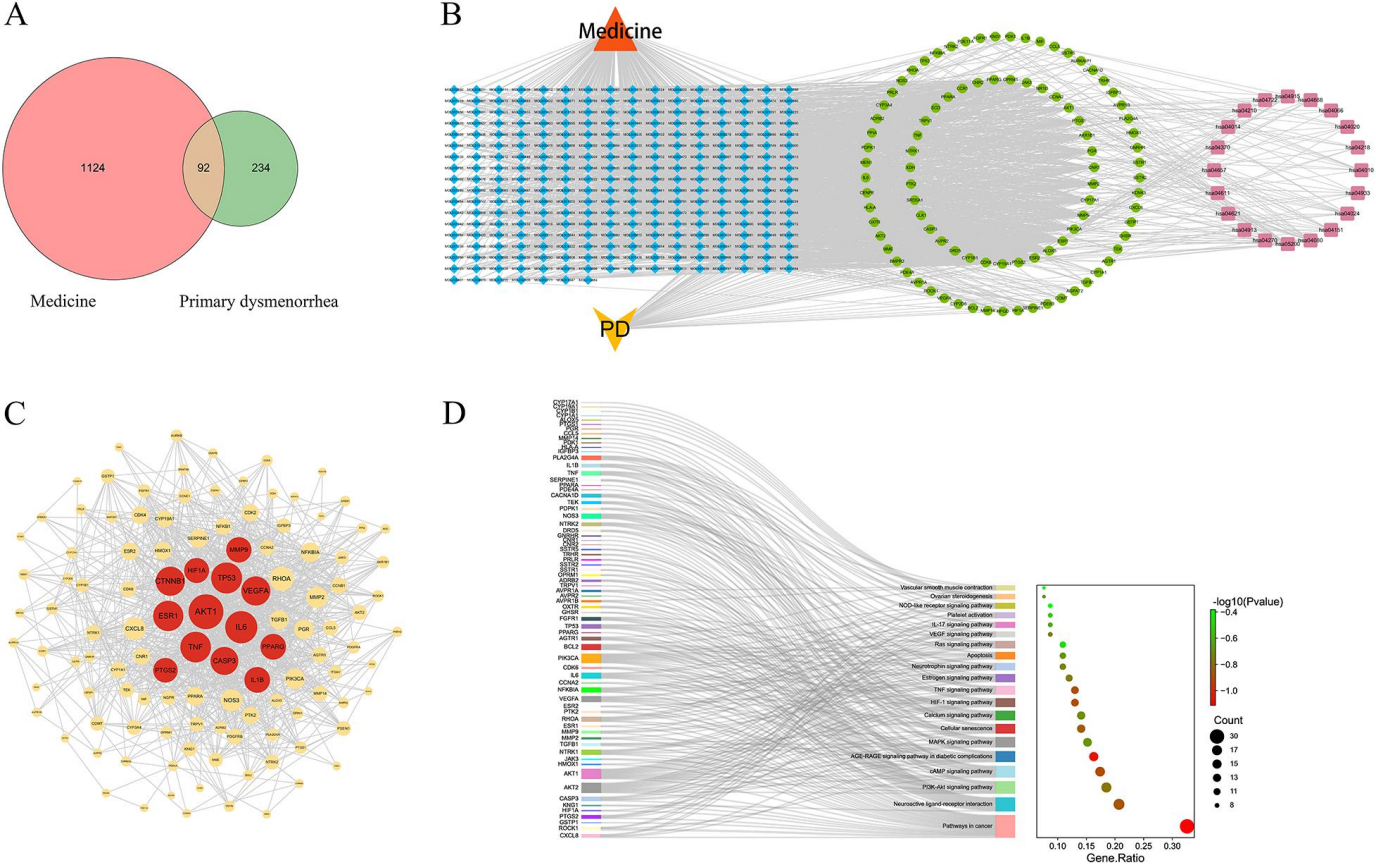
Network pharmacology predicts the multi-target mechanism of NGZT against PD. **(A)** Venn diagram of the intersection between NGZT and PD targets. **(B)** Compound-Target-Pathway network. **(C)** Protein-protein interaction (PPI) network of the common targets. **(D)** Kyoto Encyclopedia of Genes and Genomes (KEGG) pathway enrichment analysis.

After removing duplicates, 279 active compounds were retrieved from NGZT, corresponding to 1,216 predicted targets. Integration with 326 PD-related targets from disease databases yielded 92 common targets (Figure 3A), which were visualized in a compound-target-pathway interaction network (Figure 3B). This network highlighted compounds such as 16-oxo-mansumbin-3(28) and 11-Keto-beta-boswellic acid (KBA) with high topological degree values, suggesting their potential significance. To prioritize the most pivotal targets among the 92 common candidates, a dual-perspective scoring strategy was implemented. First, a PPI network was constructed, identifying 12 hub targets (e.g., AKT1, IL-6, TNF, ESR1, PTGS2) with high connectivity (degree ≥45) (Figure 3C). Concurrently, the topological significance of each target within the Compound-Target-Pathway network was assessed. A composite score was calculated by assigning equal weight (50% each) to the normalized degree from both networks. Based on the highest composite scores, CYP19A1 (aromatase), PTGS2 (prostaglandin-endoperoxide synthase 2, also known as COX-2), and ESR1 (estrogen receptor α) were ultimately selected as the three core targets for further validation (detailed scores in Supplementary Table S1).

The selection of key compounds for molecular docking was guided by both network topology and established pharmacological evidence. KBA was identified as the top compound prioritized by the network analysis. Additionally, Eugenol, Imperatorin, Paeoniflorin, and Tetrahydropalmatine—four well-characterized bioactive constituents of NGZT with documented anti-inflammatory, analgesic, or hormonal activities (see Supplementary Table S2 for their basic characteristics and sources)—were selected to illustrate the pharmacological breadth of the formulation. KEGG enrichment analysis of the 92 common targets revealed significant associations with pathways critically involved in the pathophysiology of PD, including the PI3K-Akt, MAPK, TNF, and estrogen signaling pathways (Figure 3D). This supports the hypothesis that NGZT exerts its effects through the concurrent modulation of inflammatory and hormonal networks.

### 3.3 Molecular docking validates stable binding between key compounds and core targets

**Figure 4:**
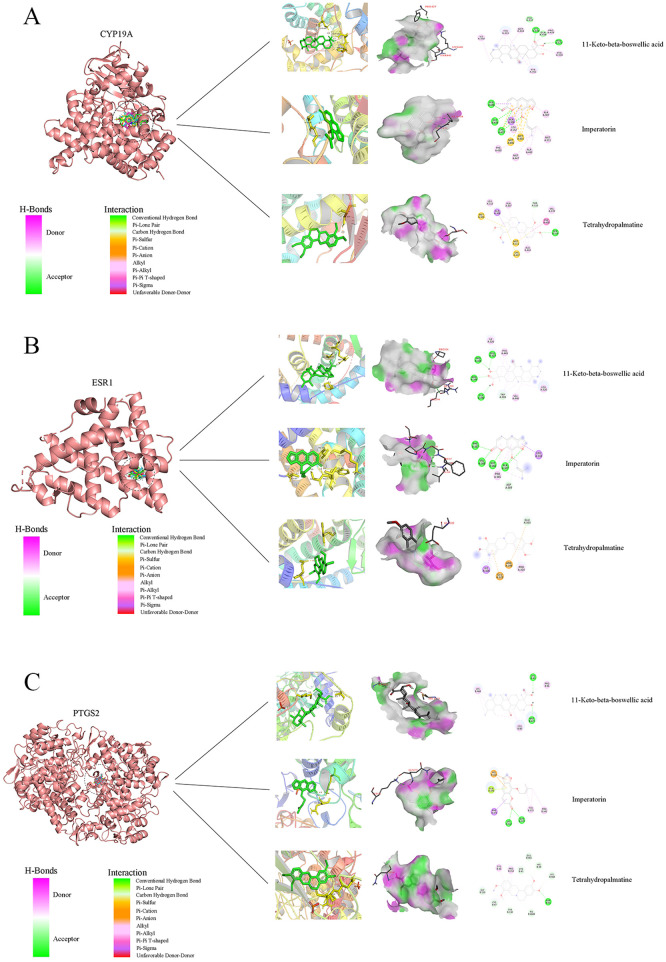
Molecular docking validation of key compound-target interactions, Two-dimensional and three-dimensional diagrams show the binding poses of representative active compounds within the active sites of core targets. **(A)** Aromatase (CYP19A1). **(B)** Estrogen receptor α (ESR1). **(C)** Prostaglandin-endoperoxide synthase 2 (PTGS2).

To computationally validate the predicted interactions, molecular docking simulations were conducted between the five selected compounds and the three core targets (CYP19A1, ESR1, PTGS2). The binding energies for all ligand-receptor complexes were favorable (below 0 kcal/mol), with approximately 78% demonstrating high-affinity interactions (less than −5.0 kcal/mol), indicating spontaneous and stable binding (Supplementary Figure S1). A detailed analysis of the binding modes through 2D and 3D diagrams (Figure 4) confirmed the formation of stable complexes via specific molecular interactions, including hydrogen bonds, π-π stacking, and hydrophobic contacts, within the active sites or key functional regions. Notably, CYP19A1, ESR1, and PTGS2 exhibited a pronounced ability to form stable conformations with the small-molecule ligands. This analysis provides a structural rationale for the proposed modulatory actions of NGZT's constituents on these pivotal PD-related targets, thereby supporting the multi-target mechanism predicted by network pharmacology.

### 3.4 Multi-omics integration elucidates coordinated regulation of genes, proteins, and metabolites

To obtain an unbiased, system-wide view of the treatment effects, we conducted transcriptomic, proteomic, and metabolomic analyses on uterine tissues.

#### 3.4.1 Transcriptomics reveals regulation of hormonal and Contractile pathways

**Figure 5:**
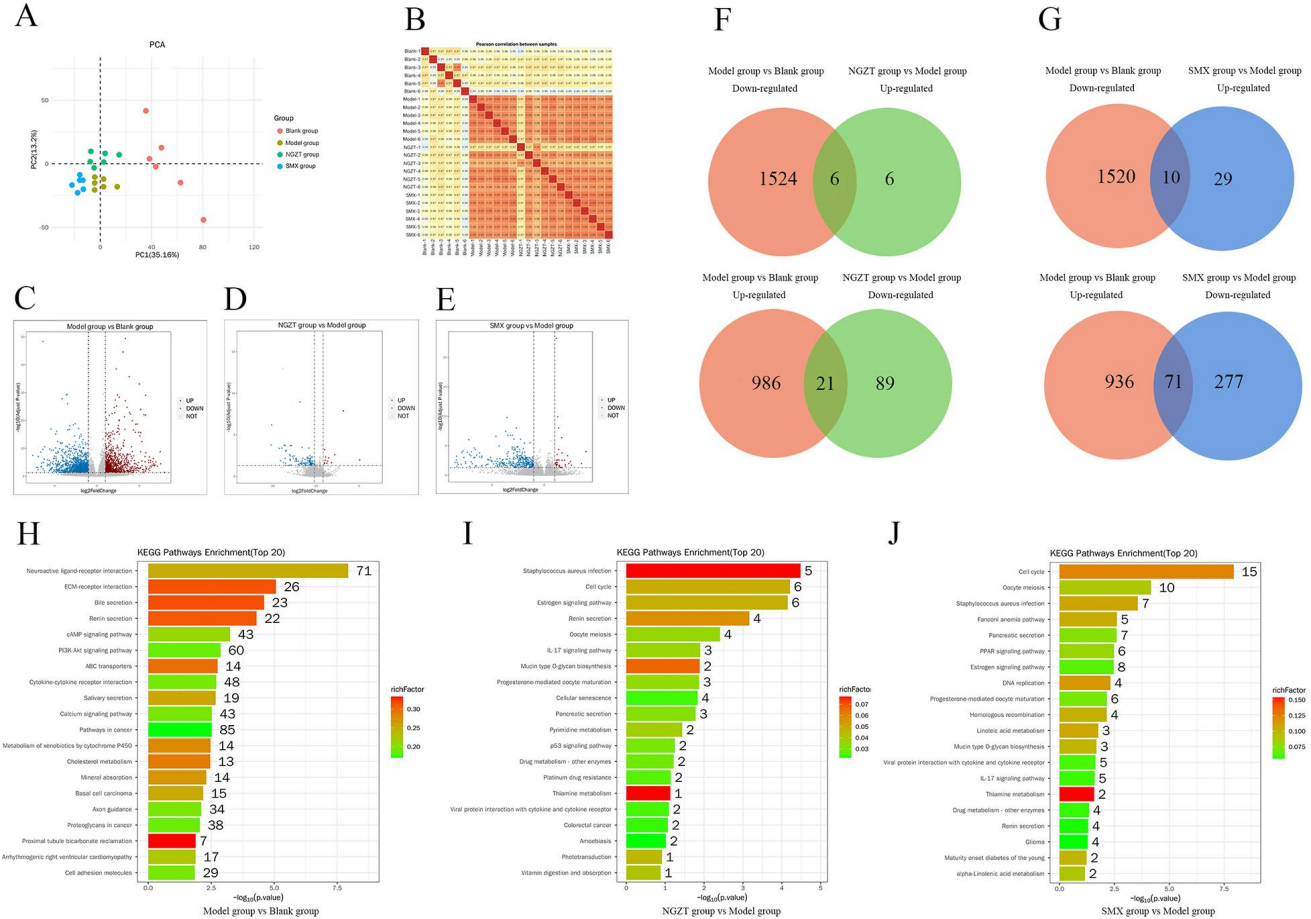
Transcriptomics analyses reveal the regulatory effects of NGZT and SMX on uterine tissues in PD rats. **(A)** Result of Principal component analysis (PCA) between groups. **(B)** Heatmap of correlation coefficients between groups. **(C)** Volcano plots of differentially expressed genes (DEGs) between Model Group and Blank group **(D)** Volcano plots of DEGs between NGZT Group and Model Group **(E)** Volcano plots of DEGs between SMX Group and Model Group. **(F)** Venn diagram of regulated genes between NGZT Group and Model Group. **(G)** Venn diagram of regulated genes between SMX Group and Model Group. **(H)** Bar plot of KEGG pathway enrichment analysis of DEGs between Model Group and Blank group **(I)** Bar plot of KEGG pathway enrichment analysis of DEGs between NGZT Group and Model Group **(J)** Bar plot of KEGG pathway enrichment analysis of DEGs between SMX Group and Model Group. DEGs were identified using thresholds of *p* *<* 0.05 and |log2(FC)| > 1.

Principal Component Analysis (PCA) and correlation analysis confirmed good data quality and inter-group separability (Figures 5A,B). Modeling induced extensive changes, with 2537 DEGs. Treatment with NGZT and SMX reversed the expression of 122 and 387 genes, respectively (Figures 5C–G, Table 4). Notably, both plasters significantly modulated the expression of Pla2g10, a gene implicated in arachidonic acid (AA) release, and Calm1, a key regulator of calcium signaling. KEGG analysis showed that the DEGs reversed by treatment were predominantly enriched in the “Estrogen signaling pathway” and “Progesterone-mediated oocyte maturation” (Figures 5H–J), supporting a link between the treatment and the regulation of sex hormone signaling.

**Table 4:** Statistical summary of differentially expressed genes (DEGs) between groups.

Comparison groups	Up-regulated	Down-regulated	Summary
Model group vs. Blank group	1,007	1,530	2,537
NGZT group vs. Model group	12	110	122
SMX group vs. Model group	39	348	387

The numbers represent the counts of genes meeting the significance thresholds of *p* *<* 0.05 and |log2(FC)| > 1.

#### 3.4.2 Proteomics confirms modulation of inflammatory and signaling proteins

**Figure 6:**
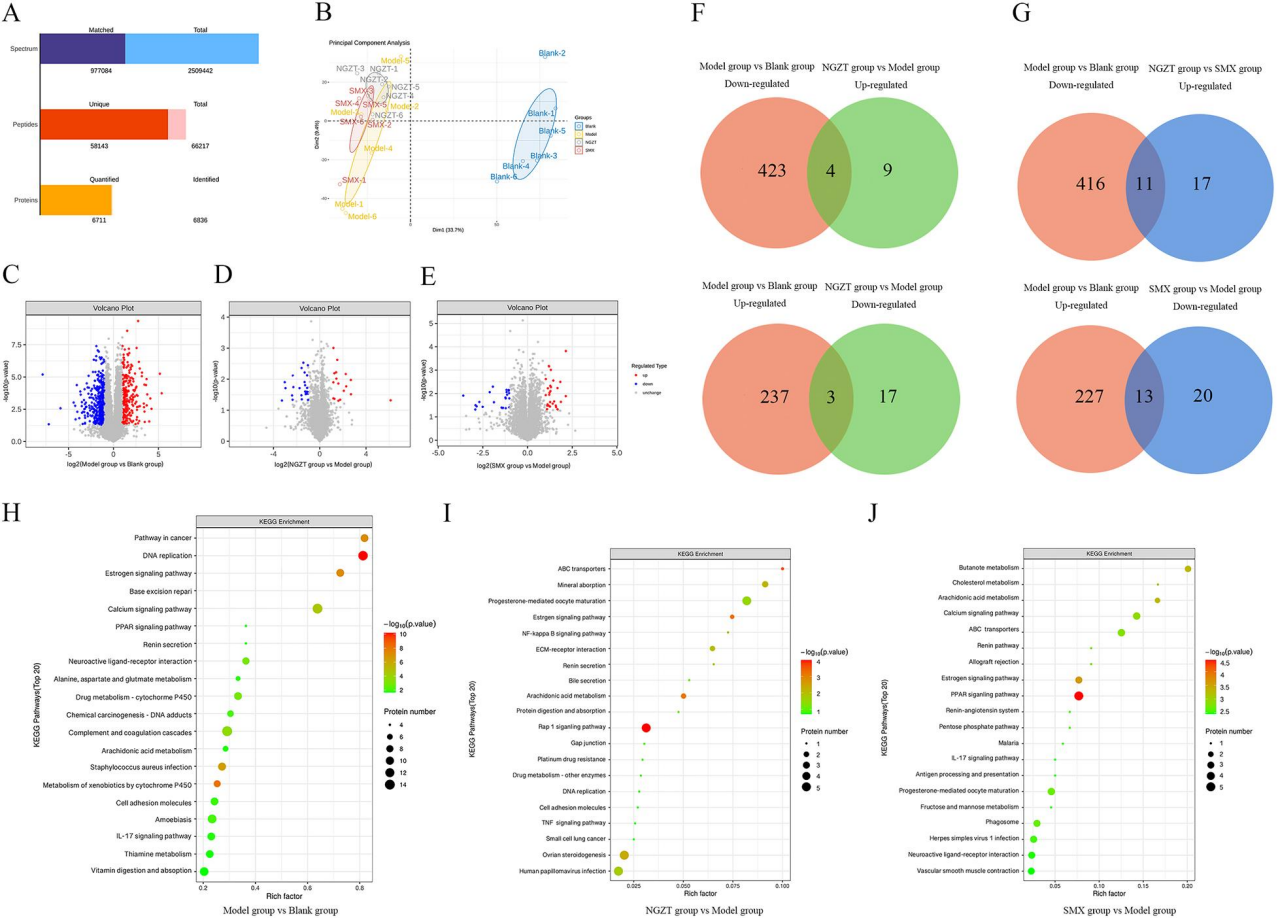
Proteomics analyses confirm protein modulation by NGZT and SMX in PD rats. **(A)** Sample data validation. **(B)** Result of PCA between groups. **(C)** Volcano plots of differentially expressed proteins (DEPs) between Model Group and Blank group. **(D)** Volcano plots of differentially DEPs between NGZT Group and Model Group. **(E)** Volcano plots of differentially DEPs between SMX Group and Model Group. **(F)** Venn diagram of regulated genes between NGZT Group and Model Group. **(G)** Venn diagram of regulated genes between SMX Group and Model Group. **(H)** Bubble plot of KEGG pathway enrichment analysis of DEPs between Model Group and Blank group. **(I)** Bubble plots of KEGG pathway enrichment analysis for DEPs between NGZT Group and Model Group. **(J)** Bubble plots of KEGG pathway enrichment analysis for DEPs between SMX Group and Model Group. DEPs were screened with criteria of *p* *<* 0.05 and |log2(FC)| > 1.

The number of spectra, peptides, and proteins in the study was counted to draw the conclusion presented in Figure 6A. The PCA results shown in Figure 6B indicated that the Blank group was completely separated from the other three groups. Although the confidence circles of the Model group, NGZT group, and SMX group samples slightly overlapped, they remained distinguishable. Proteomic analysis identified 667 DEPs in the model group. NGZT and SMX treatment reversed 33 and 61 proteins, respectively (Figures 6C–G, Table 5). Key regulated proteins included VASP (vasodilator-stimulated phosphoprotein), HSP 70 (heat shock protein 70), and MAPK1 (Mitogen-Activated Protein Kinase 1), which are involved in inflammation, cellular stress, and signal transduction. Pathway analysis of the DEPs consistently highlighted “Arachidonic acid metabolism” and the “Estrogen signaling pathway” (Figures 6H–J), corroborating the transcriptomic findings.

**Table 5:** Statistical summary of differentially expressed proteins (DEPs) between groups.

Comparison groups	Up-regulated	Down-regulated	Summary
Model group vs. Blank group	240	427	667
NGZT group vs. Model group	13	20	33
SMX group vs. Model group	28	33	61

The numbers represent the counts of proteins meeting the significance thresholds of *p* *<* 0.05 and |log2(FC)| > 1.

#### 3.4.3 Metabolomics unravels alterations in lipid and inflammatory metabolites

**Figure 7:**
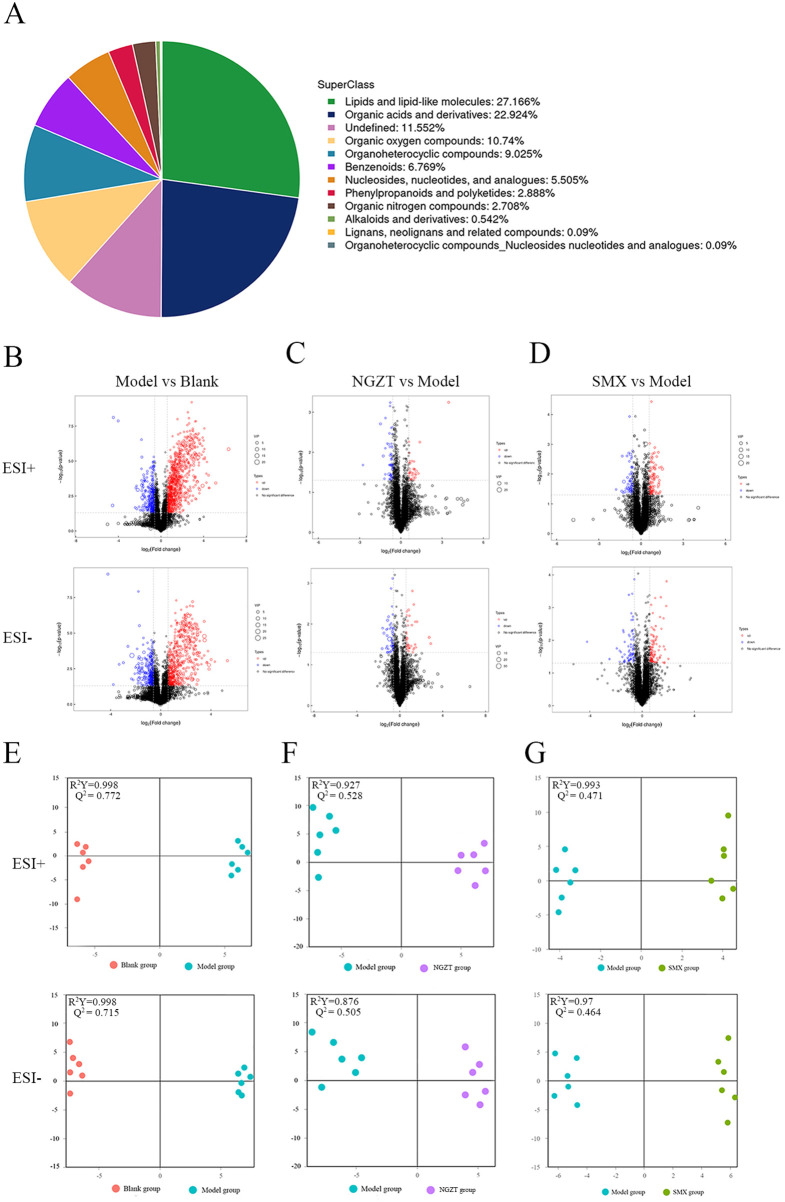
Metabolomics analyses uncover metabolic alterations regulated by NGZT and SMX. **(A)** Pie chart of identified metabolites. **(B)** Volcano plots of differential metabolites (DMs) between Model Group and Blank group. **(C)** Volcano plots of DMs between NGZT Group and Model Group. **(D)** Volcano plots of DMs between SMX Group and Model Group. **(E)** Orthogonal partial least squares-discriminant analysis (OPLS-DA) score plots for Model vs. Blank. **(F)** OPLS-DA score plots for NGZT vs. Model. **(G)** OPLS-DA score plots for SMX vs. Model. DMs were selected based on *p* *<* 0.05 and |log2(FC)| > 1.

The high reproducibility of the data was demonstrated by the overlapping total ion chromatograms and the tightly clustered quality control samples in PCA (Supplementary Figure S2). Analysis of the identified metabolites revealed that lipid and lipid-like molecules (27.17%) and organic acids and derivatives (22.92%) were the predominant classes (Figure 7A). Modeling induced significant metabolic dysregulation, yielding 101 DMs between the Model and Blank groups, with 65 up-regulated and 36 down-regulated (Table 6, Figure 7B). A notable upregulation of organic oxygen compounds was observed in the model group. Orthogonal partial least squares-discriminant analysis (OPLS-DA) demonstrated clear separation between each compared pair (Figures 7E–G), confirming the robustness of the metabolic differences.

**Table 6:** Statistical summary of differential metabolites (DMs) between groups.

Comparison groups	Up-regulated	Down-regulated	Summary
Model group vs. Blank group	65	36	101
NGZT group vs. Model group	11	21	32
SMX group vs. Model group	27	24	51

The numbers represent the counts of metabolites meeting the significance thresholds of *p* *<* 0.05 and |log2(FC)| > 1.

**Figure 8:**
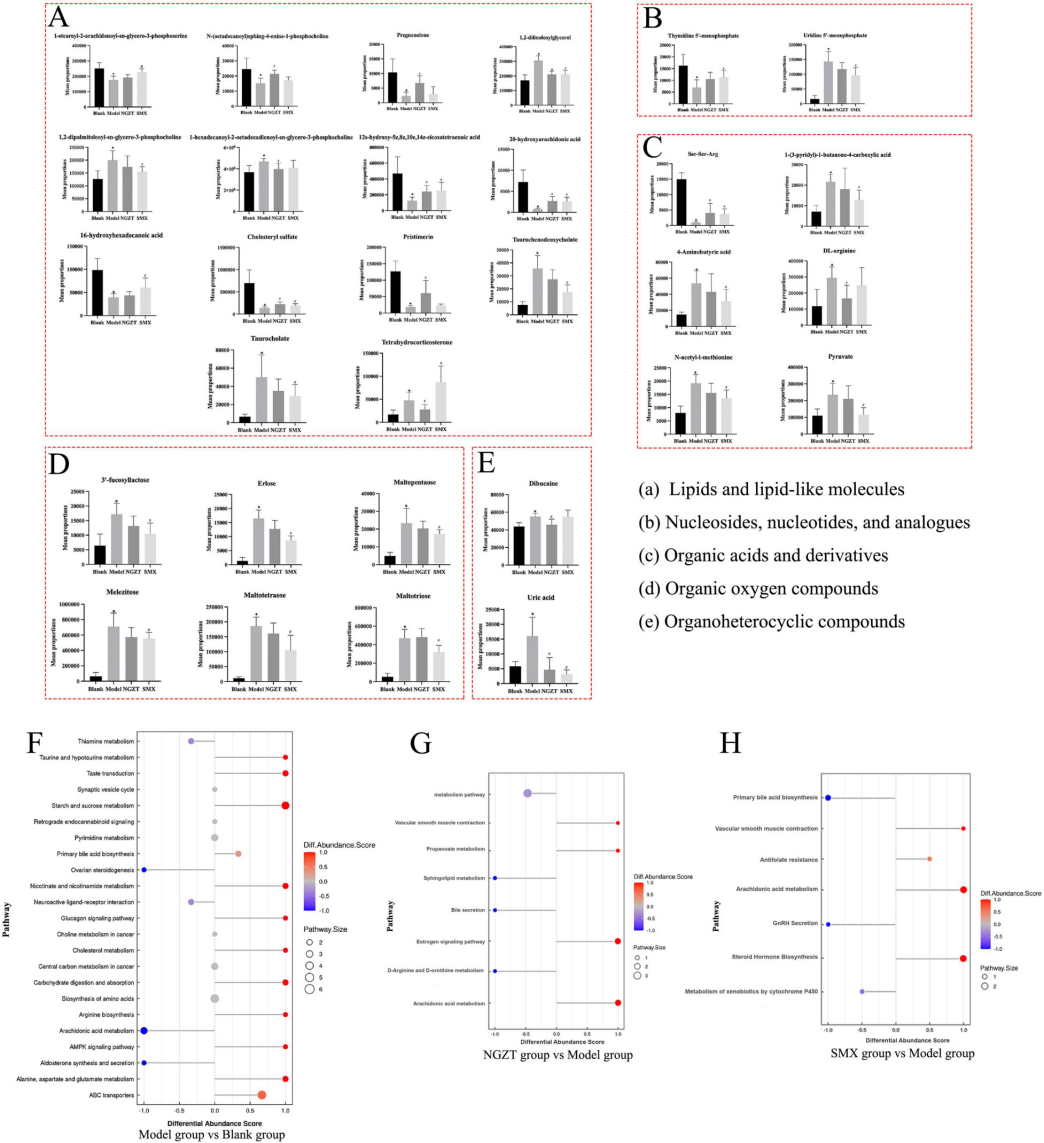
Key differential metabolites (DMs) and enriched pathways after treatment. **(A)** Metabolites belong to lipid and lipid-like molecule-like molecules. **(B)** Metabolites belong to nucleosides, nucleotides, and analogues. **(C)** Metabolites belong to organic acids and derivatives. **(D)** Metabolites belong to organic oxygen compounds. **(E)** Metabolites belong to organoheterocyclic compounds. **(F)** Bubble plot of KEGG pathway enrichment analysis of DMs between Model Group and Blank group. **(G)** Bubble plot of KEGG pathway enrichment analysis of DMs between NGZT Group and Model Group. **(H)** Bubble plot of KEGG pathway enrichment analysis of DMs between SMX Group and Model Group. * *p* *<* 0.05 compare to the blank group, ^#^ *p* *<* 0.05 compare to the model group.

Treatment with NGZT and SMX effectively reversed these alterations, regulating 32 and 51 DMs, respectively, compared to the Model group (Table 6, Figures 7C,D). A comparison of DMs across groups identified several key metabolites that changed significantly following treatment (Figures 8A–E). These included lipid mediators such as 1,2-dilinoleoylglycerol, 12S-HETE, 14Z-eicosatetraenoic acid, and 20-HETE, along with cholesteryl sulfate, the tripeptide Ser-Ser-Arg, and uric acid. Notably, SMX treatment uniquely led to a significant upregulation of six organic oxygen compounds, including 3′-fucosyllactose and erlose.

To elucidate the functional implications, KEGG pathway enrichment analysis was performed on the DMs. Differential abundance score analysis highlighted several significantly enriched pathways (Figures 8F–H), most prominently “Arachidonic acid metabolism”, “Estrogen signaling pathway”, “Vascular smooth muscle contraction”, and the “Calcium signaling pathway”.

### 3.5 NGZT and SMX inhibit inflammatory response and coagulation dysfunction

**Figure 9:**
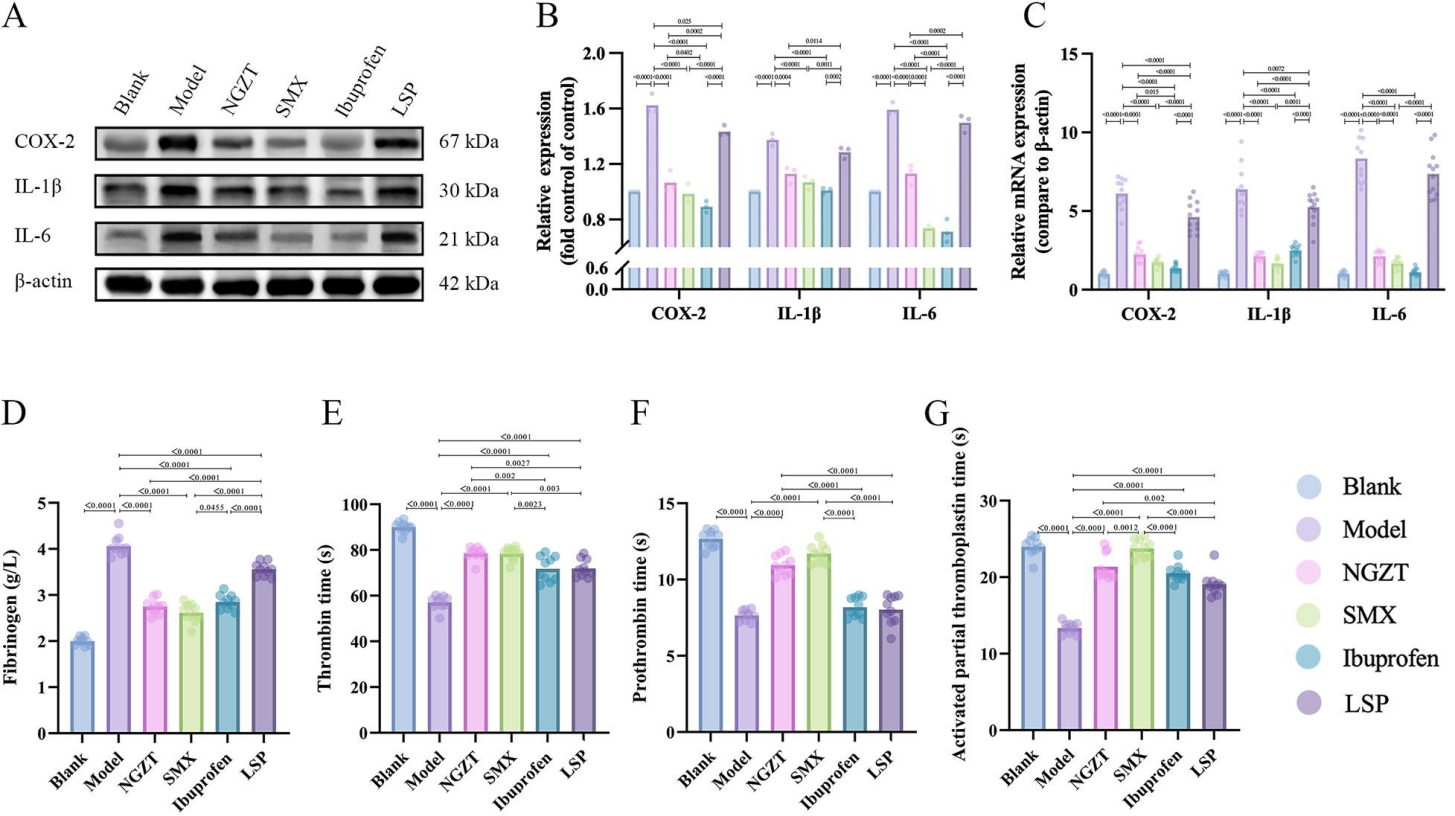
NGZT and SMX inhibit uterine inflammation and correct systemic hypercoagulability. **(A)** Representative western blot images of inflammatory proteins in uterine tissue. **(B)** Quantitative analysis of protein expression levels of COX-2, IL-6, and IL-1β. **(C)** Quantitative analysis of mRNA expression levels of COX-2, IL-6, and IL-1β. **(D)** Plasma concentration of fibrinogen (Fib). **(E)** Thrombin time (TT). **(F)** Prothrombin time (PT). **(G)** Activated partial thrombin time (APTT). Statistical significance was determined using one-way analysis of variance (ANOVA) followed by Tukey's multiple comparisons test.

Guided by the multi-omics and bioinformatics results, we validated the involvement of inflammatory pathways. Western blot and RT-qPCR analyses confirmed that the protein and mRNA levels of COX-2, IL-6, and IL-1β were significantly upregulated in the model uterus and were dramatically suppressed by NGZT, SMX, and ibuprofen treatments (Figures 9A–C). Furthermore, the PD model induced a hypercoagulable state, evidenced by increased Fib levels and shortened TT, PT, and APTT. Both TCM plasters effectively normalized these coagulation parameters (Figures 9D–G). This indicates that the anti-inflammatory action of the plasters extends to mitigating secondary coagulation abnormalities.

### 3.6 NGZT and SMX restore Sex hormone balance and regulate the HPO axis

**Figure 10:**
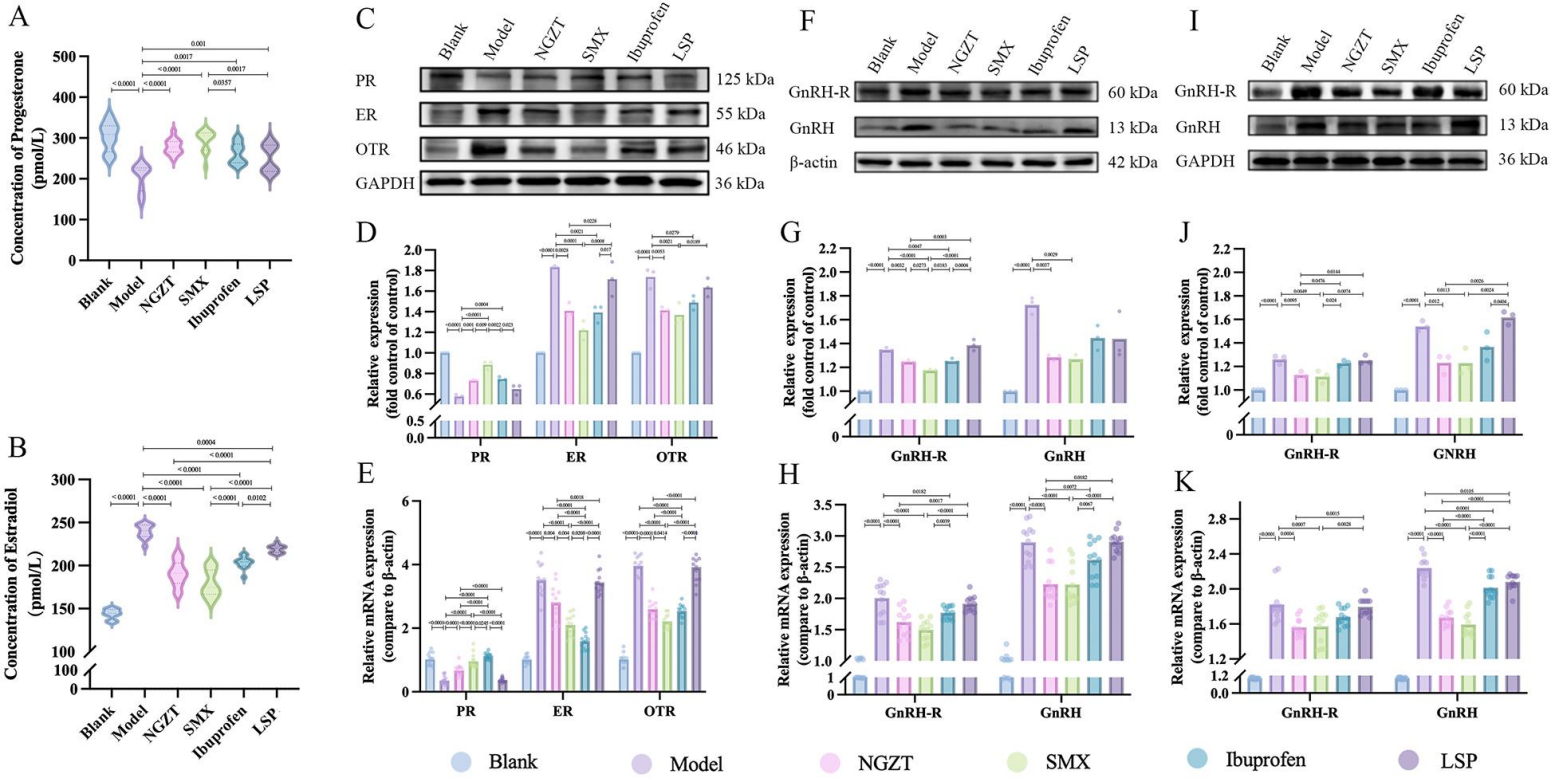
NGZT and SMX restore sex hormone balance and modulate the HPO axis. **(A)** Serum levels of progesterone in rats. **(B)** Serum levels of estradiol in rats. **(C)** Representative western blots of progesterone receptor (PR), estrogen receptor (ER), and oxytocin receptor (OTR) in the uterus. **(D)** Quantification of protein expression of PR, ER, and OTR in the uterus. **(E)** Quantification of mRNA expression of PR, ER, and OTR in the uterus. **(F)** Representative western blots of gonadotropin-releasing hormone receptor (GnRH-R) and GnRH in the ovaries. **(G)** Quantification of protein expression of GnRH-R and GnRH in the ovaries. **(H)** Quantification of mRNA expression of GnRH-R and GnRH in the ovaries. **(I)** Representative western blots of GnRH-R and GnRH in the hypothalami. **(J)** Quantification of protein expression of GnRH-R and GnRH in the hypothalami. **(K)** Quantification of mRNA expression of GnRH-R and GnRH in the hypothalami. Statistical significance was determined using one-way analysis of variance (ANOVA) followed by Tukey's multiple comparisons test.

Given the consistent enrichment of estrogen signaling in our omics data, we investigated the effects on hormonal regulation. ELISA showed that the PD model disrupted sex hormone balance, characterized by decreased serum progesterone and increased estradiol levels. Both plasters, particularly SMX, effectively reversed these changes (Figures 10A,B). In uterine tissue, the model group showed increased protein and mRNA expression of ER and OTR, and decreased PR, all of which were normalized post-treatment (Figures 10C–E). Moreover, the dysregulation extended to the HPO axis, with elevated GnRH and GnRH-R expression in both the ovaries and hypothalamus of model rats. Treatment with NGZT and SMX significantly downregulated these levels (Figures 10F–K). These results demonstrate that the plasters alleviate PD not only locally in the uterus but also by systemically rebalancing the neuroendocrine axis.

## 4 Discussion

This study demonstrates that the traditional Chinese medicine plasters NGZT and its graphene-modified counterpart SMX exert significant therapeutic effects against PD in a rat model. Through an integrated approach combining bioinformatics, multi-omics, and experimental validation, we provide compelling evidence that their efficacy stems from a multi-target mechanism, primarily involving the suppression of uterine inflammation and the restoration of sex hormone balance via modulation of the HPO axis.

### 4.1 NGZT and SMX ameliorate PD symptoms via a multi-target mechanism distinct from single-target NSAIDs

While NSAIDs like ibuprofen provide relief by primarily inhibiting COX enzymes, their associated side effects and non-response in a subset of patients highlight the need for alternative therapies (36). Our findings posit that NGZT and SMX offer a complementary approach by simultaneously targeting multiple nodes of PD pathophysiology. Network pharmacology and molecular docking predicted stable interactions between key plaster constituents (e.g., 11-Keto-beta-boswellic acid, Eugenol) and core targets like PTGS2 (COX-2), ESR1 (ER), and CYP19A1. Crucially, while the plasters downregulated COX-2 and reduced PGs—an effect shared with ibuprofen—they additionally normalized the expression of estrogen and progesterone receptors and modulated the HPO axis, effects not typically associated with NSAIDs (37, 38). This broader pharmacological profile may underlie their efficacy and potentially benefit patients who respond poorly to conventional anti-inflammatories.

### 4.2 Suppression of local uterine inflammation and systemic hypercoagulability

The PD model triggered a pronounced inflammatory response in the uterus, characterized by the upregulation of COX-2, IL-6, and IL-1β. Both NGZT and SMX potently suppressed these markers, aligning with the predicted inhibition of PTGS2. Beyond the uterus, we discovered a systemic hypercoagulable state in model rats, evidenced by elevated fibrinogen and shortened coagulation times. The ability of the plasters to normalize these parameters suggests a previously underappreciated link between PD pain and coagulation cascades, possibly mediated by inflammation-induced activation of the coagulation system (39). This anti-inflammatory and anti-hypercoagulability effect provides a robust explanation for the observed reduction in writhing behavior and uterine swelling.

### 4.3 Restoration of Sex hormone homeostasis and HPO axis function

A central finding of our multi-omics analysis was the consistent enrichment of the estrogen signaling pathway. This guided our investigation into the hormonal aspects of PD. The model successfully recapitulated a hormonal imbalance, with decreased progesterone and increased estradiol levels, a classic profile in PD pathogenesis (40). NGZT and SMX, particularly SMX, effectively reversed this imbalance. Furthermore, the plasters corrected the aberrant expression of uterine sex hormone receptors (ER, PR) and the oxytocin receptor (OTR), which are critical for mediating the actions of sex hormones and inducing uterine contractions (41–43). Most significantly, we found that the dysregulation extended to the central HPO axis, with elevated GnRH and GnRH-R in the hypothalamus and ovaries. The ability of the plasters to downregulate this overactive axis demonstrates a systemic regulatory effect, offering a mechanistic explanation for their action in rebalancing the entire reproductive endocrine network.

### 4.4 Multi-Omics integration reveals a convergent mechanism of action

**Figure 11:**
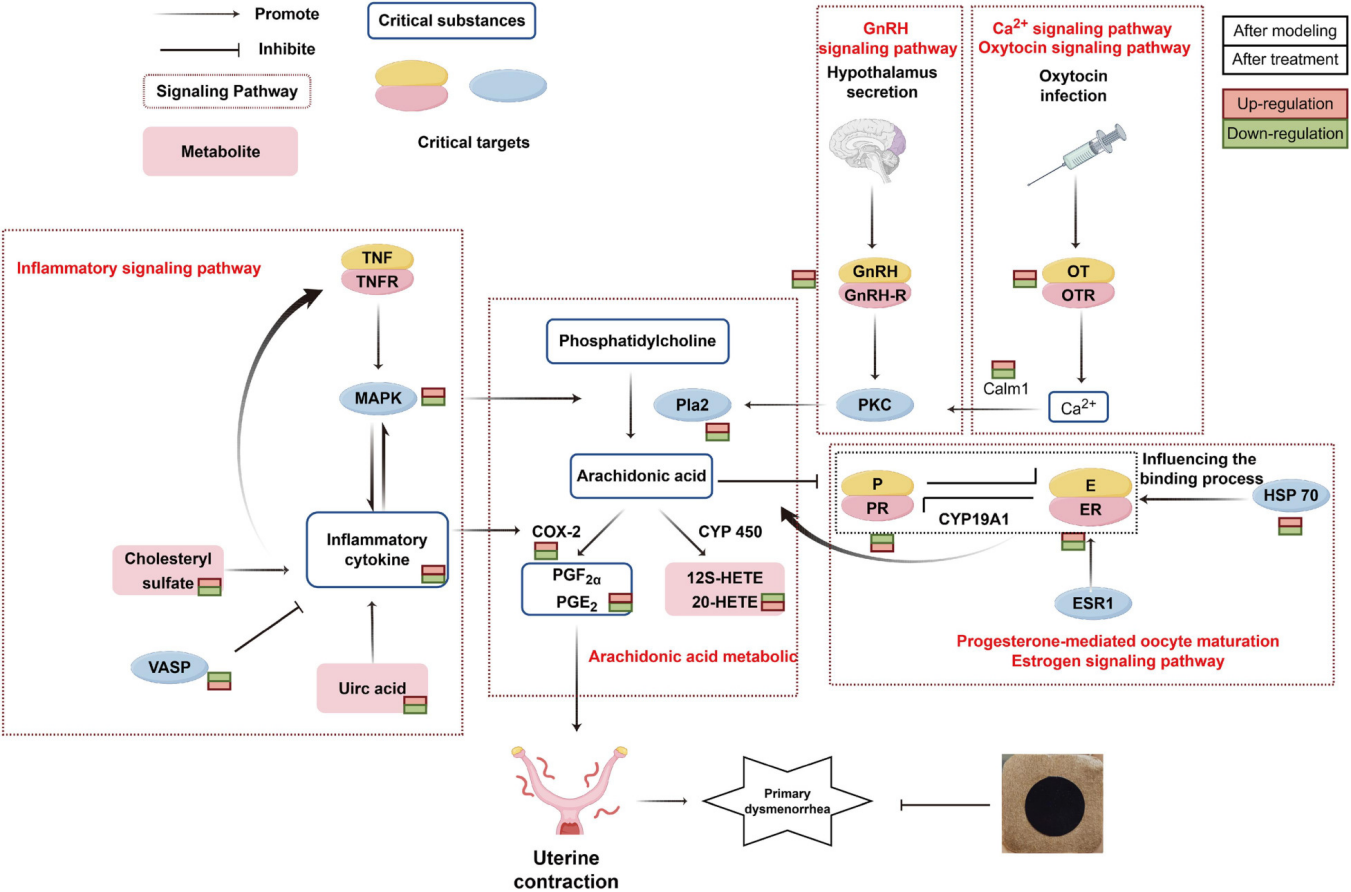
Schematic illustration of the multi-target mechanism underlying the alleviation of primary dysmenorrhea by the Chinese herbal medicine patch (SMX and NGZT). (Created with Figdraw.)

The power of our approach lies in the integration of transcriptomic, proteomic, and metabolomic data, which collectively paint a cohesive picture of the treatment effects. These multi-omics layers converged on several key pathways. First, regarding AA metabolism, transcriptomics highlighted Pla2g10, a gene responsible for releasing AA (44), while metabolomics identified a reversal in the levels of AA pathway metabolites (e.g., 12S-HETE, 20-HETE). This suggests a shunting of AA metabolism away from the pro-inflammatory COX pathway. Second, in the context of calcium signaling and smooth muscle contraction, transcriptomics identified Calm1 (Calmodulin), a key calcium sensor (45), and metabolomics enriched pathways related to “Calcium signaling” and “Vascular smooth muscle contraction”. These findings link the treatments to the direct inhibition of uterine contractility. Third, concerning cellular stress and inflammation, proteomics revealed the modulation of proteins such as HSP 70 (involved in cytoprotection) and MAPK (part of inflammatory signaling). Metabolomics further showed a reduction in oxidative stress-related organic oxygen compounds. Additionally, the regulation of VASP, which is implicated in inhibiting platelet-neutrophil complexes (46), corroborated the observed improvement in coagulation parameters. This integrated multi-omics network, summarized in Figure 11, demonstrates that NGZT and SMX do not act on a single target but rather alleviate PD by rewiring an interconnected network of genes, proteins, and metabolites.

### 4.5 The therapeutic advantage of the graphene-modified SMX plaster

Notably, SMX consistently demonstrated a superior or comparable effect to NGZT across several parameters, including the regulation of organic oxygen compounds, IL-6 expression, and HPO axis normalization. This enhanced efficacy, coupled with our finding of reduced skin irritation (Supplementary Tables S3, S4, Supplementary Figure S3), can be plausibly attributed to the incorporation of graphene. Graphene is known to improve the stability, transdermal permeability, and sustained release of drug components (47–49). This likely results in better bioavailability and more efficient engagement of the multi-target mechanisms we have elucidated. The inferior performance of the LSP patch further underscores the advantage of the complex multi-component TCM formulation over a single-agent NSAID delivered transdermally.

### 4.6 Limitations and future perspectives

The acute oxytocin-induced writhing model employed in this study elicits sustained uterine contractions and pain behaviors through exogenous hormone administration, effectively simulating the core symptoms of PD during acute episodes—namely, excessive uterine contractions and associated pain. This model has been validated for preliminary screening of analgesic efficacy (50, 51). However, it is crucial to objectively acknowledge the inherent limitations of this model: as an acute stimulation paradigm, it bypasses the natural estrous cycle and therefore cannot fully replicate the long-term neuroendocrine disturbances driven by periodic, chronic hormonal fluctuations characteristic of human PD (52). Another limitation of this study is the use of a single dosage for each therapeutic intervention. Although the selected doses for NGZT, SMX, and reference drugs were determined based on clinical equivalent conversions and preliminary pharmacodynamic data to ensure a therapeutically relevant effect, the lack of a comprehensive dose-response analysis hinders the identification of optimal therapeutic windows and the classical pharmacological validation of concentration-dependent effects. Future studies aimed at establishing detailed dose-response and time-course relationships for these plasters will be crucial for refining their clinical dosage regimens, understanding their safety profiles at varying doses, and further validating the robustness of the multi-target mechanisms identified in this study.

Nonetheless, a key objective of this study was to transcend mere analgesic phenotypes and investigate the potential pathological mechanisms underlying drug interventions. Accordingly, we systematically evaluated neuroendocrine endpoint indicators closely associated with the chronic pathophysiology of PD, particularly key molecules of the HPO axis. Notably, even following acute pain induction, model rats exhibited significant HPO axis dysfunction, including the upregulated expression of GnRH and GnRH-R in the hypothalamus and ovaries, imbalances in serum sex hormone levels (elevated estrogen and reduced progesterone), and abnormal expression of local sex hormone receptors (ER, PR) and OTR in uterine tissue. These findings suggest that acute uterine contraction stimulation, or the accompanying stress response, is sufficient to rapidly induce or unmask a series of complex neuroendocrine alterations resembling those observed in chronic PD (53).

To bridge the gap between acute models and the complex pathophysiology of human disease and to provide direction for future research, our subsequent work may proceed along two avenues. First, efforts will be directed toward developing or adopting animal models that better recapitulate the pathological features of human PD, such as the progesterone-withdrawal-induced “menstruation-like” mouse model that triggers endometrial shedding (54). Combining such models with behavioral pain assessments may establish a system capable of simulating both cyclic hormonal variations and chronic pain, thereby enabling a more comprehensive evaluation of the modulatory effects of NGZT/SMX on chronic, cyclic pathological processes. Second, clinical translational studies are essential; for example, longitudinal follow-up of PD patients with biospecimen collection across different menstrual cycle phases. Multi-omics approaches could then be applied to verify the relevance of key pathways identified in this study (e.g., arachidonic acid metabolism, estrogen signaling, and HPO axis-related molecules) in human disease, thereby assessing the validity of extrapolating mechanisms from acute animal models to chronic human pathology.

## 5 Conclusion

This study delineates the multifaceted mechanisms through which NGZT and its graphene-modified variant SMX alleviate PD. By integrating network pharmacology, multi-omics approaches, and experimental validation, we demonstrate that the therapeutic effects extend beyond merely suppressing uterine inflammation via the COX-2/PG axis to encompass the neuroendocrine system. This is achieved through the modulation of the HPO axis and the restoration of sex hormone homeostasis, while also alleviating uterine hypercontractility and associated coagulation dysfunction. The enhanced efficacy of SMX in regulating specific inflammatory and hormonal parameters may be attributed to improved transdermal delivery facilitated by graphene. Collectively, these findings provide a robust scientific foundation for the clinical application of these TCM formulations and establish a relatively systems-level framework for deciphering the complex pharmacology of multi-component natural medicines.

## Data Availability

The datasets presented in this study can be found in online repositories. The names of the repository/repositories and accession number(s) can be found in the article/Supplementary Material.
