# Supplementary material for: Integrated multi-omics elucidates the dual anti-inflammatory and neuroendocrine mechanisms of novel TCM plasters against primary dysmenorrhea
Source: Front Pain Res (Lausanne). 2026 Mar 11;7:1727963. doi: 10.3389/fpain.2026.1727963 (PMC13013513; doi:10.3389/fpain.2026.1727963)
Supplement: Supplementary file 1 [file Table1.docx]

Supplementary Material

**Supplementary Table S1**. Assignment rules and contentut.

| Rank | Target Name | Network Degree (10%) | PPI Degree (90%) | Calculation Results |
| --- | --- | --- | --- | --- |
| 1 | CYP19A1 | 132 | 22 | 77 |
| 2 | PTGS2 | 94 | 41 | 67.5 |
| 3 | ESR1 | 86 | 45 | 65.5 |
| 4 | ESR2 | 89 | 25 | 57 |
| 5 | MMP9 | 72 | 38 | 55 |
| 6 | ALOX5 | 89 | 16 | 52.5 |
| 7 | AKT1 | 48 | 56 | 52 |
| 8 | PIK3CA | 76 | 24 | 50 |
| 9 | MMP2 | 60 | 30 | 45 |
| 10 | PPARG | 39 | 40 | 39.5 |
| 11 | PGR | 53 | 26 | 39.5 |
| 12 | CYP17A1 | 69 | 9 | 39 |
| 13 | CNR1 | 55 | 22 | 38.5 |
| 14 | TNF | 26 | 50 | 38 |
| 15 | VEGFA | 20 | 47 | 33.5 |
| 16 | AKR1B1 | 51 | 13 | 32 |
| 17 | PTGS1 | 50 | 13 | 31.5 |
| 18 | PPARA | 30 | 28 | 29 |
| 19 | IL6 | 3 | 54 | 28.5 |
| 20 | CCNA2 | 43 | 12 | 27.5 |
| 21 | BCL2 | 44 | 7 | 25.5 |
| 22 | TP53 | 4 | 46 | 25 |
| 23 | CASP3 | 8 | 42 | 25 |
| 24 | JAK3 | 39 | 11 | 25 |
| 25 | IL1B | 5 | 43 | 24 |
| 26 | TRPV1 | 30 | 18 | 24 |
| 27 | HIF1A | 9 | 38 | 23.5 |
| 28 | OPRM1 | 39 | 8 | 23.5 |
| 29 | NR1I3 | 42 | 4 | 23 |
| 30 | CCR1 | 35 | 9 | 22 |
| 31 | CXCL8 | 6 | 37 | 21.5 |
| 32 | NTRK1 | 24 | 18 | 21 |
| 33 | CNR2 | 37 | 3 | 20 |
| 34 | NOS3 | 4 | 35 | 19.5 |
| 35 | RHOA | 4 | 34 | 19 |
| 36 | SCD | 30 | 6 | 18 |
| 37 | SERPINE1 | 9 | 26 | 17.5 |
| 38 | TGFB1 | 7 | 26 | 16.5 |
| 39 | PTK2 | 14 | 19 | 16.5 |
| 40 | MMP14 | 18 | 15 | 16.5 |
| 41 | HMOX1 | 6 | 24 | 15 |
| 42 | NFKBIA | 4 | 25 | 14.5 |
| 43 | CYP2D6 | 21 | 8 | 14.5 |
| 44 | AGTR1 | 7 | 21 | 14 |
| 45 | CYP1A1 | 7 | 20 | 13.5 |
| 46 | ROCK1 | 18 | 8 | 13 |
| 47 | PDE4A | 18 | 7 | 12.5 |
| 48 | AVPR1A | 19 | 6 | 12.5 |
| 49 | XDH | 19 | 6 | 12.5 |
| 50 | CCL5 | 5 | 19 | 12 |
| 51 | COMT | 8 | 16 | 12 |
| 52 | IGFBP3 | 5 | 17 | 11 |
| 53 | GSTP1 | 6 | 16 | 11 |
| 54 | TEK | 6 | 16 | 11 |
| 55 | CYP3A4 | 3 | 18 | 10.5 |
| 56 | KNG1 | 4 | 17 | 10.5 |
| 57 | AKT2 | 2 | 18 | 10 |
| 58 | CYP1B1 | 4 | 15 | 9.5 |
| 59 | PLA2G4A | 6 | 13 | 9.5 |
| 60 | MIF | 5 | 13 | 9 |
| 61 | CDK6 | 4 | 13 | 8.5 |
| 62 | ADRB2 | 3 | 13 | 8 |
| 63 | FGFR1 | 4 | 12 | 8 |
| 64 | NTRK2 | 4 | 12 | 8 |
| 65 | SRD5A1 | 10 | 5 | 7.5 |
| 66 | MME | 2 | 12 | 7 |
| 67 | GNRHR | 6 | 8 | 7 |
| 68 | HPGD | 10 | 4 | 7 |
| 69 | PRLR | 4 | 8 | 6 |
| 70 | OXTR | 3 | 8 | 5.5 |
| 71 | PDPK1 | 3 | 8 | 5.5 |
| 72 | PPIA | 3 | 8 | 5.5 |
| 73 | AVPR2 | 6 | 5 | 5.5 |
| 74 | AGPAT2 | 7 | 4 | 5.5 |
| 75 | PDE8B | 8 | 3 | 5.5 |
| 76 | AVPR1B | 5 | 5 | 5 |
| 77 | BMPR2 | 2 | 7 | 4.5 |
| 78 | MEN1 | 3 | 6 | 4.5 |
| 79 | KCNK3 | 6 | 3 | 4.5 |
| 80 | SSTR2 | 6 | 3 | 4.5 |
| 81 | PDK1 | 4 | 4 | 4 |
| 82 | GHSR | 6 | 2 | 4 |
| 83 | CACNA1D | 5 | 2 | 3.5 |
| 84 | TRHR | 5 | 2 | 3.5 |
| 85 | HLA-A | 3 | 3 | 3 |
| 86 | PDE11A | 4 | 2 | 3 |
| 87 | DRD5 | 5 | 1 | 3 |
| 88 | SSTR5 | 5 | 1 | 3 |
| 89 | CENPE | 3 | 1 | 2 |

**Supplementary TableS2.** Basic characteristics of five small-molecule ligands.

| Molecules ID | Molecule name | DL | Source | Pubchem CID |
| --- | --- | --- | --- | --- |
| MOL001259 | 11-Keto-beta-boswellic acid | 0.74 | Ruxiang(Olibanum) | 9847548 |
| MOL000254 | Eugenol | 0.04 | Dingxiang(Caryophylli Flos) | 3314 |
| MOL001941 | Imperatorin | 0.22 | Baizhi(Angelicae Dahuricae Radix) | 10212 |
| MOL001924 | Paeoniflorin | 0.79 | Baishao(Paeoniae Radix Alba) | 442534 |
| MOL004071 | Tetrahydropalmatine | 0.64 | Yanhusuo(Corydalis Rhizoma) | 72301 |

**Supplementary TableS3.** Scoring criteria for skin irritation tests

| Symptom | Classification | Mark |
| --- | --- | --- |
| Erythema | No erythema (barely visible) | 0 |
|  | Mild erythema (barely visible) | 1 |
|  | Moderate erythema | 2 |
|  | Severe erythema | 3 |
|  | Purple-red erythema and mild crusting | 4 |
| Edema | No edema | 0 |
|  | Mild edema (barely visible) | 1 |
|  | Moderate edema (visible bulge) | 2 |
|  | severe edema (Skin elevation 1mm, well defined） | 3 |
|  | severer edema (Skin elevation more than 1mm, extended) | 4 |

**Table S4.** Results of skin irritation test

| Group | | Mark | | | | Average mark |
| --- | --- | --- | --- | --- | --- | --- |
|  |  | 1h | 24h | 48h | 72h |  |
| Complete Skin | Blank group | 0.0 | 0.0 | 0.0 | 0.0 | 0.0 |
|  | NGZT group | 0.0 | 0.0 | 0.0 | 0.0 | 0.0 |
|  | SMX group | 0.0 | 0.0 | 0.0 | 0.0 | 0.0 |
| Broken skin | Blank group | 0.0 | 0.0 | 0.0 | 0.0 | 0.0 |
|  | NGZT group | 5.5 | 0.0 | 0.0 | 0.0 | 1.25* |
|  | SMX group | 0.0 | 0.0 | 0.0 | 0.0 | 0.0 |

Note:* compare to the blank group, p<0.05


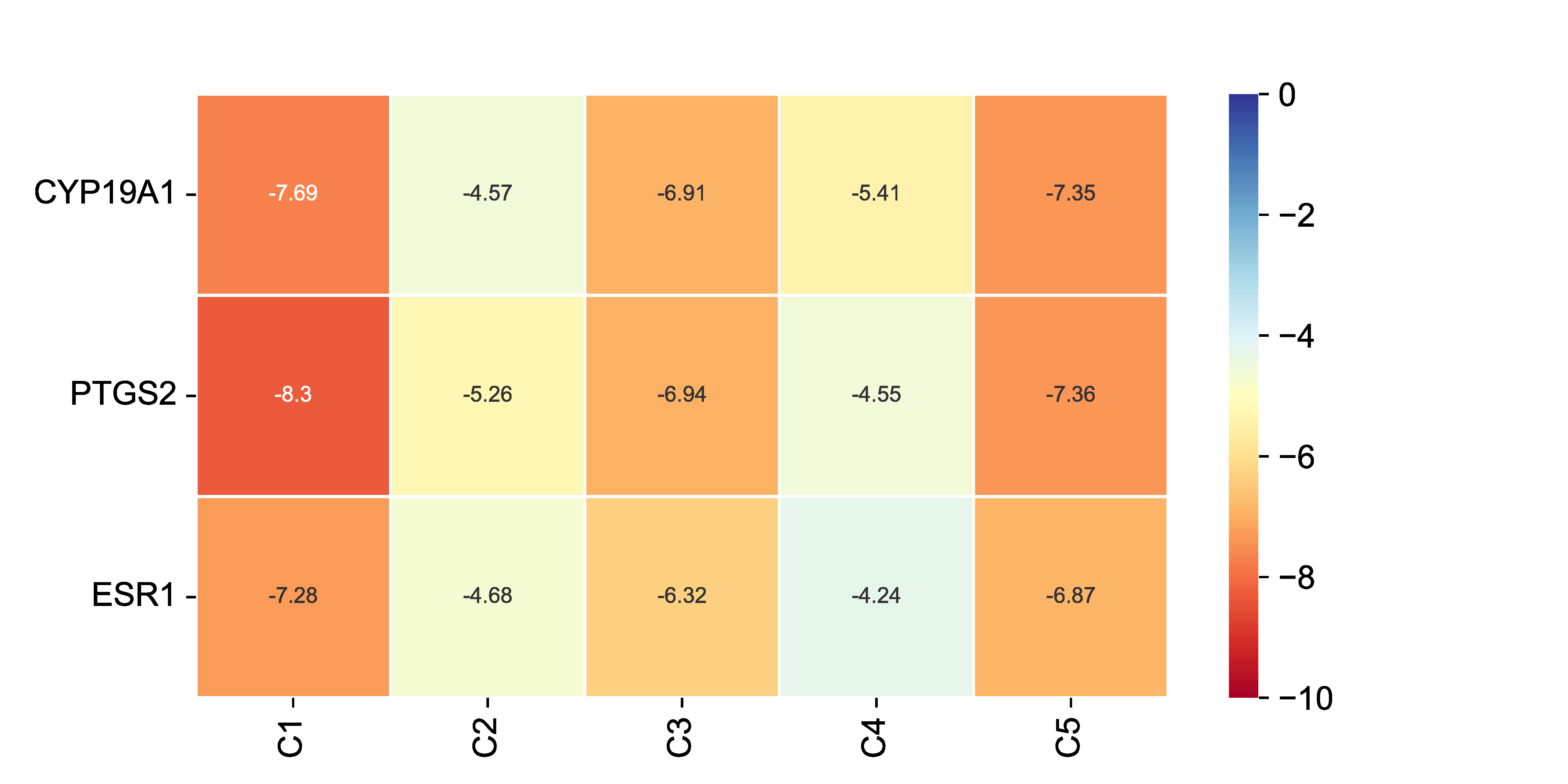


**Supplementary Figure S1.** Hotmap molecular docking of 5 core components with 3 core targets. (C1: 11-Keto-beta-boswellic acid, C2: Eugenol, C3: Imperatorin, C4: Paeoniflorin, C5: Tetrahydropalmatine). The redder the color, the stronger the docking activity.


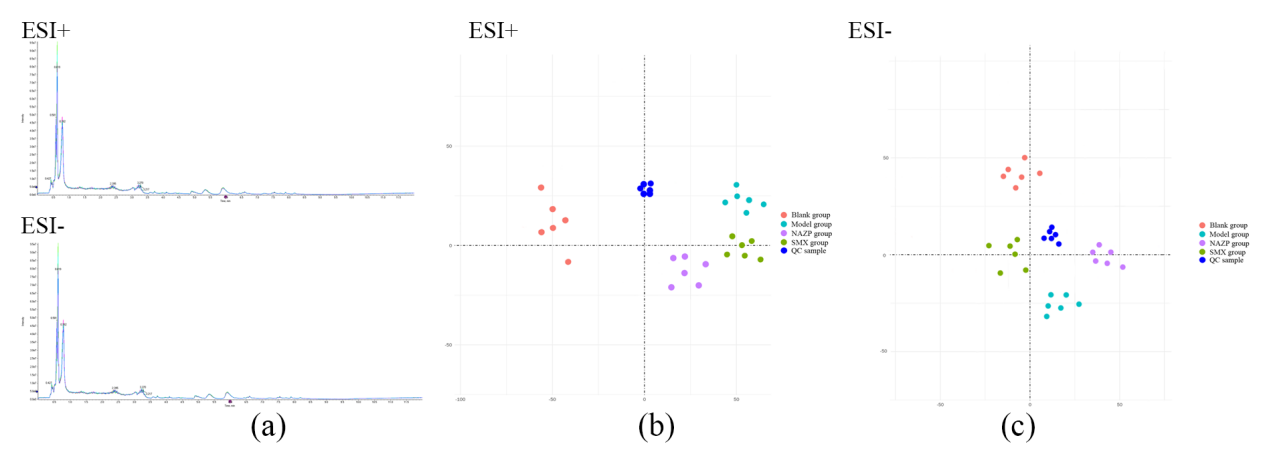


**Supplementary Figure S2.** Data validity analysis. (A) TIC of QC samples. (B) Results of PCA in positive ion mode.(C) Results of PCA in negative ion mode.


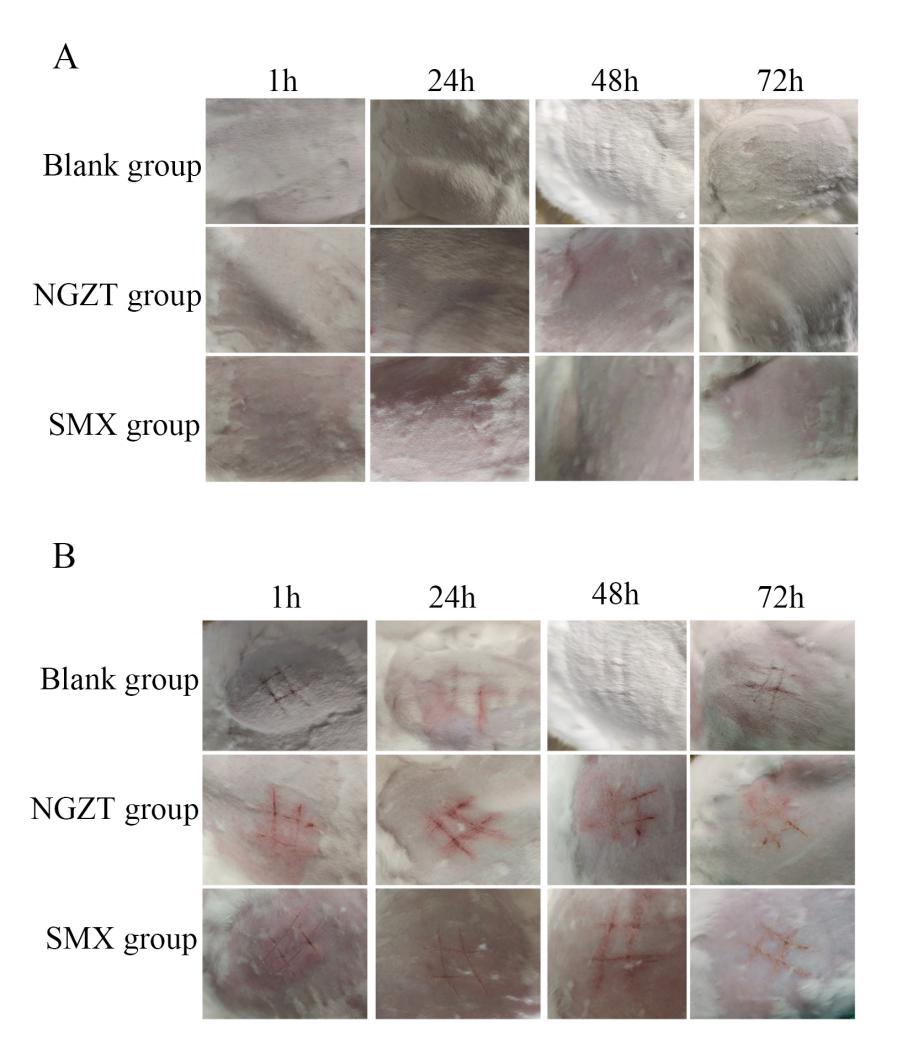


**Supplementary Figure S3.** Results of skin irritation test. (A) The irritant effects of NGZT and SMX on complete skin. (B) The irritant effects of NGZT and SMX on broken skin.
